# Assessing the Determinants of Influenza Vaccine Uptake in Malaysia: Strategies to Improve Public Health and Service Delivery

**DOI:** 10.7759/cureus.85724

**Published:** 2025-06-10

**Authors:** Prebha Manickam, Tina Varghese, Suwarna Senthilvasan, Rubithra Ramesh, Suveitra Balanei Balasubramaniam

**Affiliations:** 1 Department of Periodontology, Penang International Dental College, Butterworth, MYS

**Keywords:** health personnel, hesitancy matrix, influenza, vaccination, vaccine hesitancy

## Abstract

Background: Although the Ministry of Health, Malaysia, has emphasized that the public receive their influenza vaccine shots, with the latest announcement of free vaccines for senior citizens in Malaysia, vaccine hesitancy has been prevalent among society and more pronounced within the public who are not involved in the fields of healthcare. The present study aims to identify the influencing factors in receiving the influenza vaccine based on the vaccine hesitancy matrix proposed by the World Health Organization (WHO).

Methods: Healthcare personnel and individuals from the non-healthcare fields were enrolled. A structured questionnaire was prepared and shared by the investigators. A mixed sampling approach was employed, initially utilizing convenience sampling, and then, a respondent-driven sampling (RDS) strategy was used to target all participants consenting to fill out the questionnaire. The participants received a soft copy regarding the importance of influenza vaccination upon completion of the questionnaire.

Results: The present study showed that of the 176 participants surveyed, 80 (45.5%) were not vaccine hesitant, while 96 (54.5%) had vaccine hesitancy. Vaccine hesitancy was found to be more in number among those who are not in the healthcare sector. Having been affected by influenza previously, personal risk perception and personal vaccination experiences were found to be significant influencing factors of the willingness to receive the vaccine.

Conclusions: This study contributes to an increased understanding of the factors influencing the willingness to accept the influenza vaccine. Specific educational programs, academic-community partnerships, transparent doctor-patient communication, and involvement of policymakers could boost the rate of active immunization among the public. The medical staff are suggested to provide health education, improve doctor-patient communication, and recommend vaccinations to the public to increase their risk perception and willingness to get an influenza vaccination.

## Introduction

Reliable data from Southeast Asian countries proved that influenza is a major threat to the population’s health and well-being, with a cost impact on individuals, communities, and healthcare systems [1]. According to the World Health Organization (WHO) Virological Surveillance Summary reported to Flunet on December 18, 2024, 7392 cases were positive over 46436 specimens tested in Malaysia [2]. Without a comprehensive and consolidated body of data, these numbers are likely to be conservative, with the true burden of the disease being significantly higher. It is estimated that influenza accounts for 23% of severe acute respiratory infection (SARI) cases and 13% of pneumonia hospitalizations in Malaysia [3,4]. 

The WHO and US Centers for Disease Control and Prevention (CDC) have identified vaccination as the most effective prevention method against influenza. The influenza vaccine has been shown to reduce the spread of influenza-like illnesses (ILI) and the risk of fatal complications [5]. Retrospective studies showed a 22% reduction in ILI among Malaysian pilgrims who were vaccinated compared to unvaccinated pilgrims during the 2013 Hajj season, as well as a potential reduction in overall hospitalization rates post-vaccination [6]. There is a 55-75% vaccine effectiveness in reducing the occurrence of ILI in Malaysian adults in an old folk home, which has also been proven [7]. The cost of the influenza vaccine is Myr 41-100, which is considerably lower than the average cost for influenza treatment per year (antiviral medications/year Myr 1544) [8]. A cost-effectiveness analytical study of quadrivalent influenza vaccine (QIV) for the elderly in Malaysia compared with the no-vaccination policy showed that QIV could save over USD 4.4 million currently spent on influenza-related hospitalizations and reduce productivity losses by approximately USD 21.6 million and therefore would reduce the financial burden of managing influenza and reduce premature death related to this disease [9]. Hence, vaccination is believed to be the only stand to prevent influenza, especially in higher-risk groups, such as older adults, young children, those with comorbid conditions, healthcare workers (HCWs), and travelers.

The WHO Working Group on Vaccine Hesitancy's Strategic Advisory Group of Experts (SAGE) Report was approved in 2014, and this group defined vaccine hesitancy as "refusal or delay in acceptance of vaccination despite availability of vaccination services" [10]. Vaccine reluctance varies by time, location, and vaccine and is complicated and context-specific. Complacency, convenience, and confidence are some of the factors that influence it. Additionally, a matrix of vaccine hesitancy determinants could be used to classify specific reasons for vaccine hesitancy. This matrix included contextual influences, individual and group influences, and issues specific to vaccines [10].

Recognizing that HCWs and older adults are the two main high-risk groups, the government has significantly expanded its HCWs' vaccination coverage by means of an annual immunization program. Added to it, the latest update (February 18, 2025) reveals the nationwide influenza vaccination drive offers free influenza vaccines to senior citizens with at least one comorbidity [11].

Although the Health Ministry has emphasized the public and made measures to receive their Influenza vaccine shots, vaccine hesitancy has been prevalent among society and more pronounced within the general public who are not involved in the fields of healthcare [12]. Recent studies highlighting the concerns regarding adverse events, unduly rapid vaccine development, and poor vaccine efficacy have been controversial and have sparked some possible reasons for vaccine hesitancy among the public [13]. Considering the consistent surge in Influenza cases, fear of contracting the disease remains prominent among the high-risk community in the country. High vaccination acceptance among individuals remains a question mark, while the concern of protecting their family, society, and their own health remains prevalent [13]. The present study aims to assess the awareness, attitudes, and possible determinants of willingness to accept the influenza vaccine among the healthcare personnel and the public, and identify the influencing factors in receiving the influenza vaccine.

## Materials and methods

Study design and study population

The total duration of this study period was five months, conducted in the year 2024 in a dental school. Dental students in their clinical year of study, year 3, 4, and 5 students, dental staff, healthcare personnel, and individuals from the non-healthcare fields (n = 176) were consensually enrolled in the cross-sectional study based on the inclusion and exclusion criteria. The study included individuals aged 18 and above and individuals who could provide consent and could answer the questionnaire in English. Individuals below the age of 18, those who are not willing to participate in the study, and those who do not understand the English Language were excluded from the study.

Sampling method and sample size determination

A cross-sectional study design was employed, and participants were recruited from two primary groups: healthcare personnel and members of the public. A mixed sampling approach was employed, initially utilizing convenience sampling to recruit early participants, followed by a respondent-driven sampling (RDS) strategy to expand the participant pool through peer referrals. This approach facilitated access to a diverse sample within the constraints of available resources and timeline, which is common in behavioural health research.

Sample size estimation was conducted using G*Power version 3.1.9.4. An a priori power analysis was performed for binary logistic regression, which was the planned analysis for identifying predictors of influenza vaccine uptake. The following parameters were used: Effect size (Cohen’s f²): 0.15 (medium effect), Power (1 - β): 0.80, significance level (α): 0.05, number of predictors: 10, reflecting key constructs from the WHO's Vaccine Hesitancy Matrix, including contextual influences, individual and group influences, and vaccine-/vaccination-specific issues.

Based on these parameters, the minimum required sample size was determined to be 160 participants. This sample size is sufficient to detect statistically significant associations between the independent variables and the likelihood of receiving the influenza vaccine. The participants were informed about the purpose of the study, and participants’ consent was taken prior to participation. The questionnaire was shared on social networking websites: WhatsApp and closed group forums with the participants.

Methods of data collection

An online structured questionnaire was prepared using evidence from prior studies on willingness to receive vaccination, vaccine hesitancy in general, and influenza vaccine hesitancy among the healthcare personnel and the public [14]. The questionnaire comprised 36 closed-ended multiple-choice items and one open-ended question, organized into five thematic sections. It gathered demographic data such as gender, age, race, marital status, living arrangements, education level, residential area, and medical conditions. It also explored participants' experiences related to influenza, including prior infections, contact with infected individuals, vaccination status, and knowledge about the influenza vaccine. Additionally, the questionnaire examined participants’ concerns about vaccines and their trust in official information sources. Another focus was on their willingness to receive the influenza vaccine. Finally, it investigated the factors that influence their decision to get vaccinated, along with their perspectives on the overall importance of influenza vaccination for the community.

**Figure 1:**
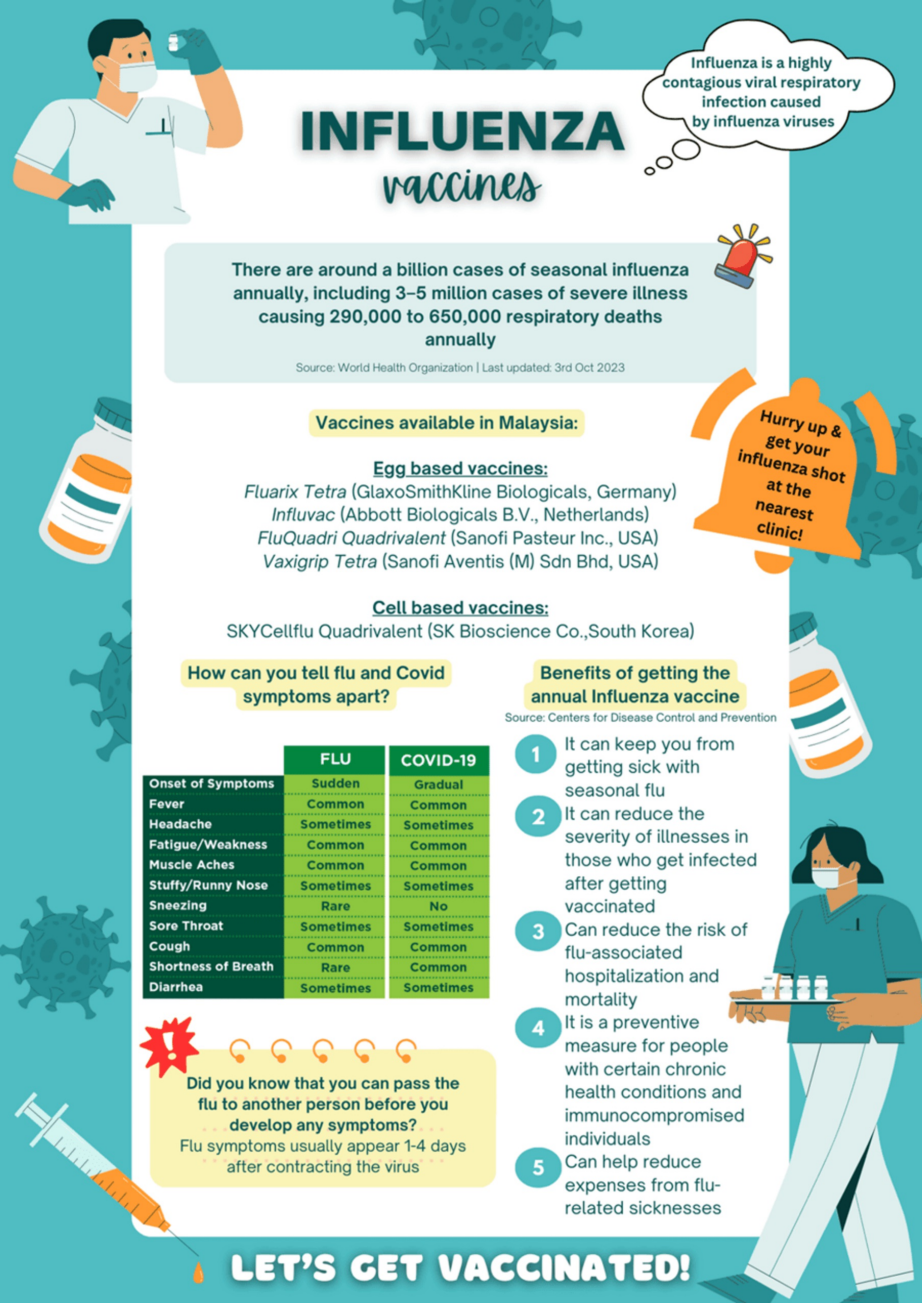
Pamphlet distributed to participants regarding the availability of influenza vaccine and its benefits

The questionnaire was prepared in the English language, which is the universal language used as the medium of instruction of most courses throughout Malaysia. The questionnaire was adapted from a similar study conducted by Zou et al., and the validity of the questionnaire was confirmed by dental specialists working in the same institution through content validity. It was conducted using the content validity index (CVI), and the question was included on a score of 3-4. An acceptable CVI score of 0.80 was obtained. It was designed to collect information regarding basic demographic details, awareness and sources of information regarding the influenza vaccine, attitudes regarding the vaccine, and prior vaccination experience. Google Forms was used to deploy this form online. Its link was shared by the investigators within the social media network of different communities, which include the healthcare personnel and the public, both individually and mainly through crowdsourcing via WhatsApp groups and closed group forums. The RDS strategy was used to target all participants who consented and were willing to spare the time to complete the survey. On the completion of the survey, the participants received a soft copy regarding the benefits of Influenza vaccination. Key insights and data presented in this pamphlet are supported by existing literature and research findings (Figure 1) [1,2,5].

Data entry and statistical analysis

Upon completion of the survey, data were downloaded and analyzed. Categorical variables related to the survey items were tabulated, and the odds ratio for vaccine hesitancy was calculated using a univariate approach. Logistic regression was conducted to test for plausible determinants of willingness to take vaccination and vaccine hesitancy while adjusting for gender, the different communities involved, and the lack of prior vaccine experience. Data collected were analyzed using IBM SPSS Statistics for Windows, Version 29 (Released 2022; IBM Corp., Armonk, New York, United States) at a significant level of p < 0.05.

Ethical consideration

The study was reviewed and approved by the Institutional Review Board (****/IRB/SRP/8/23).

## Results

Table 1 shows participant characteristics and attitudes for willingness to get vaccinated. A total of 176 valid questionnaires were collected in this survey. The majority of the participants were in the age range of 18-30 years old, accounting for 65.3%. A total of 115 female participants (65.3%) and 61 male participants (34.7%) participated in the study. This study consisted of participants from the Indian (73.9%), Chinese (11.4%), and Malay (9.1%) ethnic groups, followed by Sino-dusun, Kadazan, Punjabi, Iban, Bidayuh, Filipino, and Pakistani among other ethnic groups that collectively summed up to 5.9% of the participants involved. The proportion of healthcare personnel and individuals from the non-healthcare sector was 94 (53.4%) and 82 (46.6%), respectively.

**Table 1:** Participants’ characteristics and attitudes for willingness to vaccination N1: not willing to vaccinate; N1%: percentage of participants not willing to vaccinate; N2: willing to vaccinate; N2%: percentage of participants willing to vaccinate; N: total; N%: total percentage

General	Participant	Attitude toward vaccination				Total	Total %	Chi-square value	p-value*
Factors	Categories	N1	N1%	N2	N2%	N	N%	(χ²)	(<0.05)
Gender	Male	32	18.2	29	16.5	61	34.7	0.06	0.686
	Female	64	36.4	51	29	115	65.3		
Races	Chinese	12	12.5	8	10	20	11.4	2.718	0.534
	Indian	68	70.8	62	77.5	130	73.9		
	Malay	1.1	11.5	5	6.3	16	9.1		
	Others	3	2.9	5	5.1	10	5.9		
Marital status	Divorced	2	2.1	2	2.2	4	2.3	0.137	0.934
	Married	28	15.9	25	14.2	53	30.1		
	Single	66	37.5	53	30.1	119	67.6		
Age	18-30 years	63	35.8	52	45.2	115	29.5		
	31-40 years	14	8	9	5.1	23	13.1	6.632	0.250
	41-50	9	5.1	11	6.3	20	11.4		
	51-60	5	2.8	8	4.5	13	7.4		
	61-70	4	2.3	0	0	4	2.3		
	71-80	1	0.6	0	0	1	0.6		
Living with	Children	13	7.4	16	9.1	29	16.5	4.018	0.212
	Healthy adults	60	34.1	44	25	104	59.1		
	Living with people >65 years	5	2.8	10	5.7	15	8.5		
	Chronic diseases	8	4.5	3	1.7	11	6.3		
	Non-applicable	10	5.7	7	4	17	9.7		
Education Level	Diploma	6	3.4	7	4	13	7.4	2.147	0.709
	Secondary education	3	1.7	1	0.6	4	2.3		
	Undergraduate	59	33.5	53	30.1	112	63.6		
	Postgraduate	23	13.1	17	9.7	40	22.7		
	Others	5	2.8	2	1.1	7	4		
Healthcare	No	50	28.4	32	18.2	82	46.6	7.314	0.110
	Yes	46	26.1	68	27.3	94	53.4		
Residence	Rural	8	4.5	6	3.4	14	8	0.0	0.839
	Urban	88	50	74	42	162	92		
Immunocompromised	No	87	49.4	80	39.8	157	89.2	0.022	0.506
	Yes	9	5.1	10	5.7	19	10.8		
Family history of cancer	No	67	38.1	50	28.4	117	66.5	0.74	0.308
	Yes	29	16.5	30	17	59	33.5		
Medical insurance	Maybe	8	4.5	3	1.7	11	6.3	6.904	0.065
	No	111	6.3	3	1.7	14	8		
	Yes	77	43.8	74	42.0	151	85.8		

Table 2 shows the influencing factors associated with the willingness to receive the influenza vaccine. Previous experience with influenza, knowledge of vaccine and severity of disease, recommendations to vaccinate by friends, family, and doctors, and believing in the safety and efficacy of vaccinations had a statistically significant value. Accordingly, they have been discussed in terms of three dimensions which had an impact on the willingness to take the influenza vaccine.

**Table 2:** Influencing factors associated with willingness to receive the influenza vaccine N1: not willing to be vaccinated; N1%: percentage of participants not willing to be vaccinated; N2: willing to be vaccinated; N2%: percentage of participants willing to be vaccinated; N: total; N%: total percentage

Matrix	Factors	Opinion	Attitudes or willingness to be vaccinated				Total	Total%	Chi-square value	p-value*
Contextual influences	Questions based on hesitancy	Options	N1	N1%	N2	N2%	(N)	(N%)	(χ²)	<0.05
	Have you ever had influenza?	I don’t know	30	17	11	6.3	41	23.3	23.444	0.000
		Not infected	57	32.4	38	21.6	95	54		
		Yes, and confirmed	9	5.1	31	17.6	40	22.7		
	Have you heard any negative information about vaccines?	No	47	26.7	30	17	77	43.8	1.886	0.127
		Yes	49	27.8	50	28.4	99	56.3		
Individual and group influences	How much do you know about the influenza vaccine? (1-5)	1 (very little)	15	8.5	4	2.3	19	10.8	18.240	0.056
		2 (little)	14	8	12	6.8	26	14.8		
		3 (mod)	43	24.4	35	19.9	78	44.3		
		4 (just enough)	20	11.4	20	11.4	40	7.4		
		5 (a lot)	4	2.3	9	5.1	13	7.4		
	Influenza vaccination can help reduce the severity of the disease if I'm infected	Strongly disagree	2	1.1	3	1.7	5	2.8	18.241	0.057
		Disagree	3	1.7	4	2.3	7	4		
		Neutral	19	10.8	13	7.4	32	18.2		
		Agree	50	28.4	28	15.9	78	44.3		
		Strongly agree	22	12.5	32	18.2	54	30.7		
	Influenza vaccination can reduce my probability of being infected with the disease	Strongly disagree	3	1.7	2	1.1	5	2.8	2.773	0.210
		Disagree	4	2.3	3	1.7	7	4		
		Neutral	20	11.4	9	5.1	29	16.5		
		Agree	50	28.4	39	22.2	89	50.6		
		Strongly agree	19	10.8	27	15.3	26.1	5		
	I fear being infected by influenza	Strongly disagree	6	3.4	4	2.3	10	5.7	1.445	0.178
		Disagree	11	6.3	5	21	16	9.1		
		Neutral	31	17.6	21	11.9	52	29.5		
		Agree	40	22.7	34	19.3	74	42		
		Strongly agree	8	4.5	16	9.1	24	13.6		
	I believe the influenza vaccine is a necessity	Strongly disagree	6	3.4	4	2.3	10	5.7	1.439	0.177
		Disagree	11	6.3	5	21	16	9.1		
		Neutral	31	17.6	21	11.9	52	29.5		
		Agree	40	22.7	34	19.3	74	42		
		Strongly agree	8	4.5	16	9.1	24	13.6		
	Have you been recommended by your family, classmates, friends, or doctor to take the influenza vaccine?	No	46	26.1	13	7.4	59	33.5	18.240	0.000
		Yes	50	28.4	67	38.1	117	66.5		
	Do you trust the vaccine-related advice given by medical professionals?	No	11	6.3	5	2.8	16	9.1	3.97	0.231
		Yes	85	48.3	75	42.6	160	90.9		
	"I believe that the benefits of the influenza vaccine outweigh its possible side effects"	Strongly disagree	0	0	1	0.6	1	0.6	4.220	0.144
		Disagree	6	3.4	3	1.7	9	5.1		
		Neutral	28	15.9	19	10.8	47	26.7		
		Agree	47	26.7	33	18.8	80	45.5		
		Strongly agree	15	8.5	24	13.6	39	22.2		
	"I already got infected with influenza previously, and this gave me enough immunity"	Strongly disagree	18	10.2	14	8	32	18.2	3.99	0.273
		Disagree	16	9.1	18	10.2	34	19.3		
		Neutral	42	23.9	29	16.5	71	40.3		
		Agree	17	9.7	11	6.3	28	15.9		
		Strongly agree	3	1.7	8	4.5	11	6.3		
Vaccine-specific influences	Do you believe in the efficacy of vaccines?	No	13	7.4	3	1.7	16	9.1	3.947	0.024
		Yes	83	47.2	77	43.8	160	90.9		
	Do you believe in the safety of domestic vaccines?	No	21	11.9	9	5.1	30	17	2.773	0.052
		Yes	75	42.6	71	40.3	146	83		
	Do you believe in the safety of vaccines abroad?	No	19	10.8	8	4.5	27	15.3	1.591	0.207
		Yes	77	43.8	72	40.9	149	84.7		
	Have you been vaccinated against COVID-19?	Received 1^st^ and 2^nd^ dose	17	9.7	13	7.4	30	17		0.484
		Received 1^st^, 2^nd^ dose, and 3^rd^ dose	50	28.4	44	25	94	53.4		
		Received 1^st^, 2^nd^ dose, 3^rd^ dose, and 4^th^ dose	6	3.4	8	4.5	14	8		
		Received 1^st^, 2^nd^ dose but willing to receive 3^rd^ dose and 4^th^ dose	11	6.9	11	6.8	23	15.3		
		Received 1^st ^and 2^nd^ dose but willing to receive 3^rd^ dose and 4^th^ dose	12	6.9	3	1.7	15	8.5		
	Do you believe that the way to overcome the COVID-19 pandemic is mass vaccination?	No	33	19.3	25	14.2	59	33.5	5.660	0.736
		Yes	62	35.2	55	31.2	117			
	"I will take the influenza vaccination only if it is made mandatory for me by government authorities or the college and not on my own accord"	No	61	33.7	35	19.9	96	54.8	18.508	0.001
		Yes	60	34.1		11.4				
	Do you agree that point-of-care (POC)^1^ testing using rapid influenza diagnostic tests (RIDTs)^2^ on patients is necessary in the clinic before a dental procedure?	No	22	23.5%	33	28.9	55	48.2%	4.621	0.033
		Yes	20	17.5	39	37.2	59	51.8		

Table 3 shows the binary logistic regression to identify factors associated with vaccine hesitancy. The possibility of influenza vaccine hesitancy was lower for the participants whose profession was related to healthcare (OR = 0.441; CI: 0.198-0.981), for those who had an infection of influenza earlier (OR = 0.091; CI: 0.025-0.330), agreed to the fact that influenza vaccination can help reduce the severity of the disease if they are infected (OR = 0.148; CI: 0.028-0. 779) and recommended by friends and family (OR = 0.148; CI: 0.028-0. 779). The possibility of influenza vaccine hesitancy was higher if the participants were unwilling to take the 3rd and 4th dose of COVID-19 which was not mandated by the government unlike the 1st and 2nd doses (OR = 8.558; CI: 1.500-48.814) and was lower if they were willing to take only if it was made mandatory by the government authorities (OD = 0.547; CI = 0.361-0.831). 

**Table 3:** Binary logistic regression to identify factors associated with vaccine hesitancy OR: odds ratio; CI: confidence Interval; Rf: reference

Matrix	Factors	Reference (Rf)	Wald chi-square test	p-value	OR	95% CI	
Contextual influences	Is your profession related to healthcare?	Yes (Rf-no)	4.022	0.045	0.441	0.198	0.981
Individual and group influences	Have you ever had Influenza?	Yes (Rf-no)	13.26	0.000	0.091	0.025	0.330
	Influenza vaccination can help reduce the severity of the disease if I'm infected	Agree (Rf-disagree)	5.071	0.024	0.148	0.028	0.779
	Have you been recommended by your family, classmates, friends, or doctor to take the influenza vaccine?	Yes (Rf-no)	16.373	0.000	0.124	0.45	0.340
Vaccine-specific influences	Have you been vaccinated against COVID-19?	Taken 1^st^ and 2^nd^ doses and not willing to take/ taken 3^rd^ and 4^th^ dose	5.84	0.05	8.558	1.500	48.814
	I will take the influenza vaccination only if it is made mandatory for me by government authorities or the college and not on my own accord	Yes (Rf-no)	8.046	0.005	0.547	0.361	0.831

Of the 176 participants surveyed, 80 (45.5%) were not vaccine hesitant, while 96 (54.5%) had vaccine hesitancy. Among the healthcare and non-healthcare groups of participants, vaccine hesitancy was found to be more in number among those who are not in the healthcare sector. The odds ratio (OR) for willingness to receive vaccination was calculated as the odds of willingness divided by the odds of hesitancy. Thus, the OR is 0.694, meaning that the odds of being willing to receive the vaccine are lower compared to the odds of hesitancy. If the OR is below 1, it suggests that hesitancy is more likely than willingness in this population studied.

## Discussion

This study analyzed the current situation of willingness to receive the influenza vaccine and its influencing factors among HCWs and the public, based on the vaccine hesitancy matrix proposed by the WHO. The survey results showed that 45.4% of individuals were willing to accept the influenza vaccine, and 54.5% were not willing to be vaccinated. This outcome was in line with earlier research on influenza vaccine reluctance among China's general population [14,15]. The odds ratio was below 0.694, suggesting that hesitancy is more likely than willingness in this population studied.

According to the vaccine hesitancy matrix proposed by the WHO in 2014 [10], the present research analyzed three dimensions which had an impact on the willingness to take the influenza vaccine. The first was contextual influences, such as sociodemographic characteristics. The second was individual and group influences, such as personal risk perception, trust in medical personnel, and the influence of people around. The third was vaccine-/vaccination-specific issues, such as personal vaccination experience.

In terms of contextual influences, being a healthcare professional is a significant influencing factor. Scientific evidence identifies HCWs as being at high risk, requiring annual influenza vaccination, and this fact has been one of the main reasons for this community to accept the vaccination [16]. Recent studies done on healthcare professionals on the willingness to receive the influenza vaccine found out that they believed that the influenza vaccine is useful in distinguishing influenza symptoms from those of COVID-19, providing self-protection, and preventing cross-infection [17]. The CDC recommends that all healthcare professionals be vaccinated against influenza for three main reasons: (1) to lessen the possibility that patients will contract influenza from medical personnel, (2) to safeguard medical personnel and their families from influenza, and (3) to lower medical staff absenteeism during the influenza season, which will ultimately lower the national health service expenses [5,16]. However, in the present study, 26.1% of healthcare professionals were not willing to receive the vaccination. The study done by Betsch et al. found a few reasons as to why HCWs decline influenza vaccinations [18]. These include the fear of contracting influenza from the vaccination itself, not considering themselves to be at risk, believing that their immune system can manage a trivial disease, laziness, and false beliefs. Almutairi et al. had identified workplace practices such as encouraging and offering the vaccine, awareness of vaccination guidelines, participation in training programs about the influenza vaccine, and the type of workplace settings to be the main influencing factors among healthcare personnel [19]. A systematic review identified that there is a need for awareness among HCWs to enhance influenza vaccine uptake, and its significance highlighted the need to encourage employers to provide free or subsidized influenza vaccination at their workplaces through government policy, mandating annual flu vaccination for all, especially HCWs [20-21].

In terms of individual and group influences, having been affected by influenza previously and personal risk perception are significant influencing factors. The risk perception was an individual’s subjective judgement of disease susceptibility. It included perceived severity and probability of getting influenza. There was a significant consistency between risk perception and vaccination behavior as consistent with Zou et al. and Ebrahimi studies [14,21]. Lack of knowledge or misconceptions about influenza and influenza vaccine could affect personal risk perception and willingness to receive vaccination, as shown by the chi-square association of this study about knowledge of the vaccine (p < 0.05; 24% little knowledge; 44% moderate knowledge). However, 90.9% of the participants trusted the medical personnel and considered them the most trusted source of vaccination information. This trust was the cornerstone for maintaining confidence in vaccination among participants. Medical personnel’s knowledge of and attitudes toward the vaccine have been proven to be important determinants of their own vaccination and their recommendation of the vaccine, as concluded by a study done by Lehmann et al. [22].

In terms of vaccine-/vaccination-specific issues, logistic regressions indicated that the subjects were more likely to be vaccinated if people close to them, such as family, classmates, and friends, had recommended them for the influenza vaccine. In general, the daily life trajectory of the public was mainly at college, home, and workplace, and their awareness of diseases and preventive immunization behaviors was strongly influenced by those around them. This suggests that collective vaccination by college or community may be more effective than individual vaccination. A collective vaccination strategy improves the convenience of vaccination. Collective vaccination means uniform appointments and a fixed time and place for vaccination and active responsibility, thus reducing the rate of vaccine hesitancy [14].

On the previous note, consideration must be taken on the statement "I will take the influenza vaccination only if it is made mandatory for me by government authorities or the college and not on my own accord." This was found to be one of the main influencing factors. This stresses the need for collective vaccination to be made mandatory by the government.

From the lessons learnt from the COVID-19 response, the country now aims and is working toward the pandemic influenza preparedness (PIP) framework [23]. Having a robust pandemic influenza response relies on practicing, testing, and ultimately scaling the capacities of a robust seasonal system through therapeutics, vaccination, and diagnostics. One of the main components in this framework, according to the global influenza vaccine strategy, is preparation for better global tools, including vaccines for influenza prevention, preparedness, and response. While research toward next-generation influenza vaccine is ongoing, the current strategy for vaccine supply in a pandemic relies on seasonal influenza vaccine production being switched over to pandemic vaccine.

Academic-community partnerships can be useful for the successful provision of vaccine-related knowledge and health education on diseases to increase the public’s awareness of the importance of active immunization in the control of influenza outbreaks. This will also increase vaccine acceptance to reduce rural health disparities. Transparent communication, use of facilitators to support influenza vaccination, and public awareness of the benefits of influenza vaccination can help increase vaccination coverage among the public. Continued and pertinent health education can change people’s attitudes and behavior toward vaccination over time, leading them to make conscious decisions to take the influenza vaccine annually.

Several limitations of this study should be acknowledged. First, the use of self-reported data may have introduced response bias, as participants might have over- or underreported certain behaviors or attitudes due to social desirability or recall bias. Second, although the sample size (n = 176) is adequate for a preliminary exploration, it may limit the statistical power to detect subtle associations, particularly when analyzing subgroups by age, gender, or ethnicity. Third, given the cross-sectional nature of the study, causal relationships cannot be established. Longitudinal studies are needed to confirm these associations over time. This study sample predominantly consisted of Indian participants (73.9%) and individuals aged 18-30 years (65.3%), which may limit the generalizability of the findings to other ethnic or age groups. This demographic skew indicates potential selection bias, possibly due to the sampling method or recruitment platform. Future studies should aim to include a more representative population.

## Conclusions

This study showed that the intention of individuals in the non-healthcare sectors to receive the influenza vaccine was low. It also contributes to an increased understanding of the factors influencing the willingness to accept the influenza vaccine. The influencing factors were analyzed in three dimensions. Firstly, among sociodemographic characteristics, being a healthcare personnel was a significant influencing factor as this community was more accepting of vaccinating against influenza due to their nature of work, which increases their chance of getting the illness. Secondly, among individual and group influences, having been affected by influenza previously and personal risk perception were found to be significant influencing factors. Lack of knowledge or misconceptions about influenza and influenza vaccine could affect personal risk perception and willingness to receive vaccination, as shown by the chi-square association of this study about the knowledge of the vaccine. Last but not the least, among the vaccine-/vaccination-specific issues was the personal vaccination experience. The awareness of diseases and preventive immunization behaviors were found to be strongly influenced by those around them, as individuals were more likely to be vaccinated if people close to them, such as family, classmates, and friends, had recommended them for the influenza vaccine. The following measures can be recommended. First, to improve patients' perceptions of risk and desire to have an influenza vaccination, medical personnel are advised to offer health education, enhance doctor-patient contact, and recommend immunizations. In addition, educational campaigns should be held periodically to allay misconceptions about influenza and the influenza vaccine. Finally, improving access to vaccines, removal of administrative and financial barriers to vaccination, role modeling, and monitoring the vaccination coverage with future longitudinal studies could bring in a change in improving vaccination delivery.

## Figures and Tables

**Figure 2:**
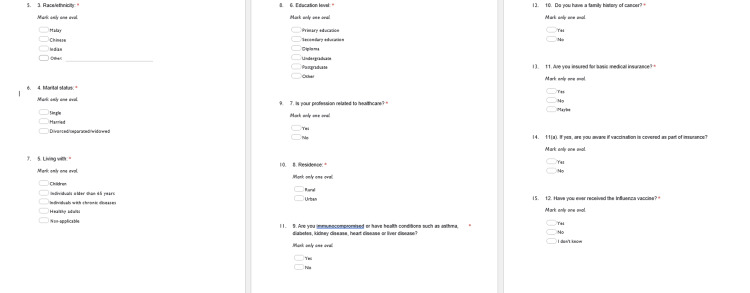
Questionnaire page 1

## Appendices

**Table 4:** Response for the questionnaire

Timestamp	Email address	I am aware that the information given in the questionnaire can be used in this study	1. Age	2. Gender	3. Race/ethnicity	4. Marital status	5. Living with	6. Education level	7. Is your profession related to healthcare?	8. Residence	9. Are you immunocompromised or have health conditions such as asthma, diabetes, kidney disease, heart disease or liver disease?	10. Do you have a family history of cancer?	11. Are you insured for basic medical insurance?	11 (a). If yes, are you aware if vaccination is covered as part of insurance?	12. Have you ever received the Influenza vaccine?	13. Have you ever had Influenza?	13 (a). If you answered yes to the above question, state the duration of your previous influenza infection	14. Influenza infection in social close contact	15. How much do you know about the influenza vaccine	16. Have you been recommended by your family, classmates, friends, or doctor to take the influenza vaccine?	17. General views regarding the importance of influenza vaccination for the community. (a) "I fear of being infected by influenza"	17. General views regarding the importance of influenza vaccination for the community. (b) "Influenza vaccination can reduce my probability of being infected by the disease"	17. General views regarding the importance of influenza vaccination for the community. (c) "Influenza vaccination can reduce the spread of the disease in the community"	17. General views regarding the importance of influenza vaccination for the community. (d) "Influenza vaccination can help reduce the severity of the disease if I'm infected"	17. General views regarding the importance of influenza vaccination for the community. (e) "I believe the influenza vaccine is a necessity"	17. General views regarding the importance of influenza vaccination for the community. (f) "I believe that the benefits of the influenza vaccine outweighs its possible side effects"	17. General views regarding the importance of influenza vaccination for the community. (g) "I already got infected with influenza previously and this gave me enough immunity"	18. Have you heard any negative information about vaccines?	18 (a). If you answered yes to the above question, what is the negative information that you've heard?	19. Concern regarding vaccines and trust of official information. (a) Do you trust the vaccine-related advice given by medical professionals?	19. Concern regarding vaccines and trust of official information. (b) Do you believe the efficacy of vaccines?	19. Concern regarding vaccines and trust of official information. (c) Do you believe the safety of domestic vaccines?	19. Concern regarding vaccines and trust of official information. (d) Do you believe the safety of vaccines abroad?	20. "I will take the influenza vaccination only if it is made mandatory for me by government authorities or college and not on my own accord'	21. The cost of the influenza vaccination affects my decision of whether to receive the vaccine or not	22. Have you been vaccinated against COVID-19?	22 (a). Why are you unwilling to receive the COVID-19 third or fourth booster?	23. "Do you believe that the way to overcome the COVID-19 pandemic is mass vaccination"	24. (Question is only for clinical students and doctors) Do you agree that Point-of-care (POC) testing using rapid influenza diagnostic tests (RIDTs) on patients is necessary in the clinic before a dental procedure?	24(a). If your answer was 4 or 5 in the above question, why do you think taking the rapid influenza diagnostic tests (RIDTs) in the clinic is necessary?					
Timestamp	selvakumari8600@gmail.com	Agree	41-50 years old	Female	Indian	Married	Healthy adults	Other	No	Urban	No	No	Yes	No	No	Not infected		Yes and confirmed	3	Yes	Neutral	Neutral	Neutral	Neutral	Disagree	Neutral	Disagree	Yes	Vaccines are dangerous/harmful and can cause serious side effects	No	No	No	No	4	4	Received 1st, 2nd, and 3rd dose	I'm worried about the side effects of the booster shots	2	4	Helps us monitor the current rate of infection among the patients					
Timestamp	kvinvictor7@gmail.com	Agree	18-30 years old	Male	Indian	Single	Healthy adults	Undergraduate	No	Urban	No	No	Yes	Yes	Yes	Yes and confirmed	1 week	I don't know	3	Yes	Agree	Agree	Agree	Agree	Agree	Agree	Agree	No	Vaccines are unnecessary as our body can fight off diseases on its own	Yes	Yes	Yes	Yes	4	4	Received 1st, 2nd, and 3rd dose		5	5	It gives me a sense of security knowing that the patient is not infected					
Timestamp	sughee2012@gmail.com	Agree	18-30 years old	Female	Indian	Single	Healthy adults	Undergraduate	No	Urban	No	No	Yes	Yes	Yes	Not infected		Not infected	3	Yes	Agree	Agree	Agree	Agree	Agree	Disagree	Disagree	Yes	Vaccines are dangerous/harmful and can cause serious side effects	Yes	Yes	Yes	No	3	3	Received 1st, 2nd, and 3rd dose	I feel that the first and second doses are already sufficient to keep me protected from the virus	5	3						
Timestamp	kumarjr99@gmail.com	Agree	18-30 years old	Male	Indian	Single	Healthy adults	Undergraduate	Yes	Urban	No	No	Yes	No	I don't know	Not infected		I don't know	3	No	Neutral	Disagree	Agree	Disagree	Agree	Disagree	Disagree	No	Vaccines are used for government control	Yes	Yes	Yes	Yes	2	4	Taken only 1st and 2nd dose but willing to receive 3rd and 4th dose	I'm worried about the side effects of the booster shots	3	3	If the patient is tested positive, I can plan an alternate treatment option					
Timestamp	rubithra1746@gmail.com	Agree	18-30 years old	Female	Indian	Single	Healthy adults	Undergraduate	Yes	Urban	No	No	Yes	No	No	I don't know		Yes and confirmed	1	Yes	Agree	Agree	Agree	Agree	Agree	Agree	Agree	Yes	Vaccines are dangerous/harmful and can cause serious side effects	Yes	Yes	Yes	Yes	5	2	Received 1st, 2nd, and 3rd dose	I don't think it's necessary as the number of COVID-19 cases has gone down already	5	3	Helps us monitor the current rate of infection among the patients					
Timestamp	varschinivasu@gmail.com	Agree	18-30 years old	Female	Indian	Single	Healthy adults	Undergraduate	Yes	Urban	No	Yes	Yes	No	I don't know	Not infected		Yes and confirmed	3	No	Neutral	Agree	Agree	Agree	Agree	Agree	Neutral	No		Yes	Yes	Yes	Yes	3	3	Received 1st, 2nd, 3rd, and 4th dose	I'm worried about the side effects of the booster shots	3	3	If the patient is tested positive, I can plan an alternate treatment option					
Timestamp	eastedele@gmail.com	Agree	18-30 years old	Female	Bidayuh	Single	Healthy adults	Undergraduate	Yes	Urban	Yes	No	Maybe	No	I don't know	I don't know		Not infected	3	Yes	Neutral	Strongly agree	Strongly agree	Strongly agree	Strongly agree	Strongly agree	Agree	No		Yes	Yes	Yes	Yes	4	1	Taken only 1st, 2nd, and 3rd dose but willing to receive 4th dose		5	4	It gives me a sense of security knowing that the patient is not infected					
Timestamp	abiramimagendran18@gmail.com	Agree	18-30 years old	Female	Indian	Single	Healthy adults	Undergraduate	Yes	Urban	No	No	Yes	Yes	Yes	Not infected		Not infected	4	Yes	Agree	Strongly agree	Strongly agree	Strongly agree	Strongly agree	Strongly agree	Strongly disagree	No		Yes	Yes	Yes	Yes	1	1	Received 1st, 2nd, and 3rd dose	There’s no option in MySejahtera to book an appointment for the fourth dose now	4	3						
Timestamp	thavyndra@gmail.com	Agree	18-30 years old	Male	Indian	Single	Healthy adults	Undergraduate	No	Urban	No	Yes	Yes	Yes	Yes	Not infected		Not infected	2	Yes	Neutral	Agree	Agree	Agree	Agree	Agree	Disagree	Yes	Vaccines are dangerous/harmful and can cause serious side effects	Yes	Yes	Yes	Yes	3	2	Received 1st, 2nd, and 3rd dose		4							
Timestamp	qiaoteng1@gmail.com	Agree	18-30 years old	Female	Chinese	Single	Healthy adults	Undergraduate	No	Urban	No	No	Yes	No	I don't know	Not infected		Not infected	3	No	Strongly agree	Agree	Agree	Agree	Neutral	Agree	Disagree	No		Yes	Yes	Yes	Yes	2	2	Received 1st, 2nd, and 3rd dose	I'm worried about the side effects of the booster shots	4							
Timestamp	npeiyi56@gmail.com	Agree	18-30 years old	Female	Chinese	Single	Non-applicable	Undergraduate	Yes	Urban	No	No	Yes	Yes	Yes	Not infected		Not infected	3	Yes	Agree	Strongly agree	Strongly agree	Strongly agree	Strongly agree	Strongly agree	Neutral	No		Yes	Yes	Yes	Yes	1	1	Taken only 1st, 2nd, and 3rd dose but willing to receive 4th dose		3	4	If the patient is tested positive, I can plan an alternate treatment option					
Timestamp	jsmnsfr@gmail.com	Agree	18-30 years old	Female	Malay	Single	Healthy adults	Undergraduate	Yes	Urban	No	No	Yes	Yes	Yes	Not infected		Not infected	2	No	Neutral	Neutral	Neutral	Neutral	Neutral	Neutral	Neutral	Yes	All of the above	Yes	Yes	Yes	Yes	4	3	Received 1st, 2nd, and 3rd dose	I feel that the first and second doses are already sufficient to keep me protected from the virus	4	4	It gives me a sense of security knowing that the patient is not infected					
Timestamp	shanmugamashresha@gmail.com	Agree	18-30 years old	Female	Indian	Single	Non-applicable	Undergraduate	No	Urban	No	No	Yes	No	I don't know	Not infected		Not infected	2	No	Agree	Agree	Agree	Agree	Agree	Agree	Agree	No		Yes	Yes	Yes	Yes	3	4	Received 1st, 2nd, and 3rd dose		5	5	Helps us monitor the current rate of infection among the patients					
Timestamp	forgetme0706@gmail.com	Agree	18-30 years old	Male	Chinese	Single	Healthy adults	Postgraduate	Yes	Urban	No	Yes	Yes	No	Yes	I don't know		I don't know	2	Yes	Neutral	Strongly agree	Strongly agree	Strongly agree	Agree	Agree	Disagree	Yes	Vaccines are dangerous/harmful and can cause serious side effects	Yes	Yes	Yes	Yes	3	5	Taken only 1st, 2nd and 3rd dose but willing to receive 4th dose		5	3						
Timestamp	thannusweetie@gmail.com	Agree	18-30 years old	Female	Indian	Single	Non-applicable	Undergraduate	Yes	Urban	No	Yes	Maybe	No	Yes	Yes and confirmed	1 week plus	I don't know	3	Yes	Strongly agree	Strongly agree	Strongly agree	Strongly agree	Strongly agree	Strongly agree	Neutral	No		Yes	Yes	Yes	Yes	1	3	Received 1st, 2nd, and 3rd dose	I feel that the first and second doses are already sufficient to keep me protected from the virus	4	4	It gives me a sense of security knowing that the patient is not infected					
Timestamp	thenussha4@gmail.com	Agree	18-30 years old	Female	Indian	Single	Healthy adults	Undergraduate	No	Urban	No	No	Yes	Yes	Yes	Yes and confirmed	1 week	Yes and confirmed	4	Yes	Agree	Neutral	Neutral	Disagree	Neutral	Neutral	Disagree	Yes	Vaccines are dangerous/harmful and can cause serious side effects	Yes	Yes	Yes	Yes	2	2	Received 1st and 2nd dose		2							
Timestamp	poojakathiravan00@gmail.com	Agree	18-30 years old	Female	Indian	Single	Healthy adults	Undergraduate	Yes	Urban	No	Yes	Yes	Yes	No	I don't know		Yes and confirmed	3	Yes	Agree	Strongly agree	Strongly agree	Strongly agree	Agree	Agree	Neutral	No		Yes	Yes	Yes	Yes	2	2	Received 1st and 2nd dose		4	4	If the patient is tested positive, I can plan an alternate treatment option					
Timestamp	hemeshwari@gmail.com	Agree	18-30 years old	Female	Indian	Single	Healthy adults	Undergraduate	Yes	Urban	No	No	Yes	No	Yes	Not infected		Yes and confirmed	4	Yes	Agree	Agree	Agree	Agree	Strongly agree	Agree	Neutral	No		Yes	Yes	Yes	Yes	2	3	Received 1st, 2nd, 3rd, and 4th dose		4	3						
Timestamp	hirani469@gmail.com	Agree	18-30 years old	Female	Indian	Single	Healthy adults	Undergraduate	No	Urban	No	Yes	Yes	Yes	I don't know	Yes and confirmed	Four days	I don't know	2	No	Agree	Strongly agree	Strongly agree	Strongly agree	Strongly agree	Neutral	Disagree	Yes	Vaccines are dangerous/harmful and can cause serious side effects	Yes	Yes	Yes	Yes	3	3	Received 1st, 2nd, and 3rd dose		5							
Timestamp	lakshanaravi05@gmail.com	Agree	18-30 years old	Female	Indian	Single	Healthy adults	Postgraduate	No	Urban	Yes	No	Yes	No	No	I don't know		I don't know	5	Yes	Agree	Agree	Agree	Agree	Agree	Agree	Neutral	Yes	Vaccines are dangerous/harmful and can cause serious side effects	Yes	Yes	Yes	Yes	1	3	Received 1st, 2nd, and 3rd dose	Done	3							
Timestamp	nramnh@gmail.com	Agree	18-30 years old	Female	Malay	Single	Healthy adults	Undergraduate	Yes	Urban	No	No	Yes	No	I don't know	Not infected		I don't know	2	No	Strongly agree	Agree	Agree	Strongly agree	Agree	Agree	Agree	Yes	Vaccines are dangerous/harmful and can cause serious side effects	Yes	Yes	Yes	Yes	5	4	Received 1st, 2nd, and 3rd dose		4	4	It gives me a sense of security knowing that the patient is not infected					
Timestamp	nandakumaranselvakumaran@gmail.com	Agree	18-30 years old	Male	Indian	Single	Healthy adults	Postgraduate	No	Urban	No	No	Yes	No	Yes	Yes and confirmed	One week	Yes and confirmed	2	No	Strongly agree	Agree	Agree	Agree	Strongly agree	Neutral	Strongly agree	Yes	Vaccines are unnecessary as our body can fight off diseases on its own	Yes	Yes	Yes	Yes	4	4	Received 1st, 2nd, 3rd, and 4th dose	I don't think it's necessary as the number of COVID-19 cases has gone down already	4	4	It gives me a sense of security knowing that the patient is not infected					
Timestamp	suveitra10balanei@gmail.com	Agree	18-30 years old	Female	Indian	Single	Healthy adults	Undergraduate	Yes	Urban	No	Yes	Yes	No	No	Yes and confirmed	4 days	Yes and confirmed	3	Yes	Agree	Agree	Agree	Agree	Agree	Agree	Strongly disagree	No		Yes	Yes	Yes	Yes	3	3	Received 1st, 2nd and 3rd dose		4	3						
Timestamp	maithilly.thurairaj@gmail.com	Agree	18-30 years old	Female	Indian	Single	Healthy adults	Other	No	Rural	No	No	Maybe	Yes	No	Not infected		I don't know	3	Yes	Disagree	Disagree	Strongly disagree	Neutral	Strongly disagree	Disagree	Neutral	Yes	Vaccines are unnecessary as our body can fight off diseases on its own	No	Yes	No	Yes	3	5	Taken only 1st and 2nd dose but willing to receive 3rd and 4th dose	I'm worried about the side effects of the booster shots	3	4	If the patient is tested positive, I can plan an alternate treatment option					
Timestamp	m-9824329@moe-dl.edu.my	Agree	18-30 years old	Female	Indian	Single	Healthy adults	Secondary education	No	Rural	No	No	No	No	No	Not infected		Not infected	1	Yes	Agree	Agree	Agree	Agree	Agree	Agree	Strongly disagree	Yes	Vaccines are dangerous/harmful and can cause serious side effects	Yes	Yes	Yes	Yes	4	4	Received 1st and 2nd dose	I'm worried about the side effects of the booster shots	3							
Timestamp	shrutal.production@gmail.com	Agree	41-50 years old	Female	Indian	Married	Children	Postgraduate	No	Urban	No	No	Yes	No	Yes	Yes and confirmed		Yes and confirmed	5	Yes	Strongly agree	Strongly agree	Strongly agree	Strongly agree	Strongly agree	Strongly agree	Strongly agree	Yes	Vaccines are dangerous/harmful and can cause serious side effects	Yes	Yes	Yes	Yes	1	3	Received 1st, 2nd, and 3rd dose	I'm worried about the side effects of the booster shots	3							
Timestamp	geethapk65@gmail.com	Agree	51-60 years old	Female	Indian	Married	Children	Postgraduate	No	Urban	No	Yes	Yes	No	Yes	I don't know		I don't know	4	Yes	Strongly agree	Agree	Agree	Strongly agree	Agree	Strongly agree	Disagree	Yes	Vaccines are dangerous/harmful and can cause serious side effects	Yes	Yes	Yes	Yes	3	3	Received 1st, 2nd, and 3rd dose	I'm worried about the side effects of the booster shots	4							
Timestamp	sanjettathanasekaran@gmail.com	Agree	18-30 years old	Female	Indian	Single	Healthy adults	Postgraduate	No	Rural	No	No	Yes	No	No	Not infected		Not infected	3	No	Agree	Agree	Agree	Agree	Neutral	Agree	Neutral	No		Yes	Yes	Yes	Yes	3	4	Received 1st, 2nd, and 3rd dose	I'm worried about the side effects of the booster shots	4	3						
Timestamp	lallinair@yahoo.com	Agree	61-70 years old	Female	Indian	Married	Healthy adults	Other	No	Urban	No	Yes	Yes	Yes	I don't know	Not infected		Not infected	1	No	Neutral	Neutral	Neutral	Neutral	Neutral	Neutral	Neutral	No		No	No	No	No	5	5	Received 1st, 2nd, 3rd, and 4th dose		3	4	Helps us monitor the current rate of infection among the patients					
Timestamp	soozhen8@gmail.com	Agree	18-30 years old	Male	Chinese	Single	Healthy adults	Undergraduate	Yes	Urban	No	No	Yes	Yes	Yes	Not infected		I don't know	3	Yes	Disagree	Agree	Agree	Neutral	Agree	Agree	Strongly disagree	Yes	Vaccines are dangerous/harmful and can cause serious side effects	Yes	Yes	Yes	Yes	2	3	Received 1st, 2nd, and 3rd dose	I don't think it's necessary as the number of COVID-19 cases has gone down already	4	3						
Timestamp	ptraj19761976@gmail.com	Agree	41-50 years old	Male	Indian	Married	Children	Undergraduate	Yes	Urban	No	No	Yes	Yes	No	Not infected		Yes and confirmed	3	Yes	Agree	Neutral	Neutral	Agree	Neutral	Neutral	Disagree	Yes	Vaccines are dangerous/harmful and can cause serious side effects	No	Yes	No	No	5	1	Received 1st and 2nd dose	I don't think it's necessary as the number of COVID-19 cases has gone down already	2							
Timestamp	yunqingtan217@gmail.com	Agree	18-30 years old	Female	Chinese	Single	Healthy adults	Undergraduate	Yes	Urban	No	No	Yes	No	Yes	Not infected		Yes and confirmed	3	Yes	Agree	Agree	Agree	Agree	Agree	Agree	Neutral	No		Yes	Yes	Yes	Yes	4	2	Received 1st and 2nd dose	I don't think its necessary as the number of COVID-19 cases has gone down already	3	3						
Timestamp	arrunhbz17@gmail.com	Agree	18-30 years old	Male	Indian	Single	Healthy adults	Diploma	No	Urban	Yes	No	Yes	Yes	No	Not infected		Not infected	1	No	Agree	Agree	Agree	Agree	Agree	Agree	Strongly disagree	Yes	Vaccines are dangerous/harmful and can cause serious side effects	Yes	Yes	Yes	Yes	1	1	Unwilling to receive 3rd dose (Please proceed to question 22a)	I don't think it's necessary as the number of COVID-19 cases has gone down already	5							
Timestamp	hansikakaur202@gmail.com	Agree	18-30 years old	Female	Punjabi	Single	Healthy adults	Undergraduate	Yes	Urban	No	No	Yes	No	I don't know	I don't know		I don't know	1	No	Neutral	Neutral	Agree	Agree	Strongly agree	Agree	Neutral	Yes	Vaccines are dangerous/harmful and can cause serious side effects	No	No	No	No	4	1	Received 1st, 2nd, and 3rd dose		3	4	If the patient is tested positive, I can plan an alternate treatment option					
Timestamp	chanaryblight@gmail.com	Agree	18-30 years old	Female	Filipino	Single	Healthy adults	Undergraduate	Yes	Rural	No	No	Yes	Yes	Yes	Yes and confirmed	1 week	I don't know	5	Yes	Neutral	Strongly agree	Agree	Strongly agree	Agree	Agree	Neutral	No		Yes	Yes	Yes	Yes	2	1	Received 1st, 2nd, and 3rd dose		5	4	If the patient is tested positive, I can plan an alternate treatment option					
Timestamp	arasi_2795@hotmail.com	Agree	18-30 years old	Female	Indian	Single	Healthy adults	Undergraduate	Yes	Urban	No	No	Yes	Yes	Yes	Yes and confirmed		Yes and confirmed	4	Yes	Agree	Agree	Agree	Agree	Agree	Agree	Neutral	Yes	Vaccines are unnecessary as our body can fight off diseases on its own	Yes	Yes	Yes	Yes	2	5	Received 1st, 2nd, 3rd, and 4th dose		4	5	If the patient is tested positive, I can plan an alternate treatment option					
Timestamp	abixis16@gmail.com	Agree	18-30 years old	Female	Indian	Single	Individuals with chronic diseases	Undergraduate	Yes	Urban	No	No	Yes	No	I don't know	I don't know		I don't know	3	Yes	Neutral	Agree	Agree	Agree	Neutral	Agree	Disagree	Yes	Vaccines are dangerous/harmful and can cause serious side effects	Yes	Yes	Yes	Yes	4	3	Taken only 1st, 2nd, and 3rd dose but willing to receive 4th dose		4	3						
Timestamp	pmdds19106061@mahsastudent.edu.my	Agree	18-30 years old	Male	Chinese	Single	Non-applicable	Undergraduate	Yes	Urban	No	Yes	Yes	No	I don't know	I don't know		I don't know	4	No	Agree	Strongly agree	Strongly agree	Strongly agree	Agree	Strongly agree	Neutral	Yes	Vaccines are dangerous/harmful and can cause serious side effects	Yes	Yes	Yes	Yes	1	3	Taken only 1st, 2nd, and 3rd dose but willing to receive 4th dose		5	3						
Timestamp	khayrinamin@gmail.com	Agree	18-30 years old	Female	Malay	Single	Healthy adults	Undergraduate	Yes	Urban	No	Yes	Yes	No	Yes	Not infected		Not infected	2	Yes	Agree	Agree	Agree	Agree	Agree	Agree	Strongly disagree	Yes	Vaccines are dangerous/harmful and can cause serious side effects	Yes	Yes	Yes	Yes	1	2	Received 1st, 2nd, and 3rd dose		4	4	If the patient is tested positive, I can plan an alternate treatment option					
Timestamp	hamizahmanaff95@gmail.com	Agree	18-30 years old	Female	Pakistani	Single	Healthy adults	Undergraduate	Yes	Urban	Yes	Yes	Yes	No	No	Not infected	None	Yes and confirmed	3	No	Agree	Agree	Agree	Agree	Agree	Agree	Strongly disagree	Yes	Vaccines are dangerous/harmful and can cause serious side effects	Yes	Yes	Yes	Yes	3	4	Received 1st, 2nd, and 3rd dose	None	3	4	If the patient is tested positive, I can plan an alternate treatment option					
Timestamp	mkmahesh021296@gmail.com	Agree	18-30 years old	Male	Indian	Single	Healthy adults	Undergraduate	No	Urban	No	No	Yes	Yes	No	Yes and confirmed		Yes and confirmed	2	Yes	Agree	Agree	Agree	Agree	Agree	Agree	Agree	No		Yes	Yes	Yes	Yes	4	2	Received 1st, 2nd, and 3rd dose		5							
Timestamp	hanushaams@gmail.com	Agree	18-30 years old	Female	Indian	Single	Healthy adults	Postgraduate	No	Urban	No	No	Yes	No	No	Not infected		Not infected	3	No	Agree	Strongly agree	Strongly agree	Strongly agree	Agree	Agree	Strongly disagree	No		Yes	Yes	Yes	Yes	3	4	Received 1st, 2nd, and 3rd dose		4							
Timestamp	qiqitan1123@gmail.com	Agree	18-30 years old	Female	Chinese	Single	Non-applicable	Undergraduate	Yes	Urban	No	No	Yes	Yes	Yes	Yes and confirmed	1 week	Yes and confirmed	3	Yes	Strongly agree	Strongly agree	Strongly agree	Strongly agree	Strongly agree	Agree	Neutral	Yes	Vaccines are dangerous/harmful and can cause serious side effects	Yes	Yes	Yes	Yes	5	5	Received 1st, 2nd, and 3rd dose	I'm worried about the side effects of the booster shots	5	5	If the patient is tested positive, I can plan an alternate treatment option					
Timestamp	kavithaanjalithanabal@gmail.com	Agree	18-30 years old	Female	Indian	Single	Individuals older than 65 years	Undergraduate	Yes	Urban	No	No	Yes	Yes	Yes	Not infected		Not infected	2	No	Disagree	Disagree	Strongly disagree	Strongly disagree	Strongly disagree	Strongly disagree	Disagree	No		Yes	Yes	Yes	Yes	5	3	Taken only 1st, 2nd, and 3rd dose but willing to receive 4th dose		5	4	It gives me a sense of security knowing that the patient is not infected					
Timestamp	sushantini16@gmail.com	Agree	18-30 years old	Female	Indian	Single	Healthy adults	Undergraduate	Yes	Urban	No	No	Yes	Yes	I don't know	Not infected		I don't know	2	No	Agree	Strongly agree	Strongly agree	Strongly agree	Strongly agree	Strongly agree	Disagree	No		Yes	Yes	Yes	Yes	4	3	Received 1st, 2nd, and 3rd dose		4	5	If the patient is tested positive, I can plan an alternate treatment option					
Timestamp	drprebsmanik@gmail.com	Agree	31-40 years old	Female	Indian	Married	Children	Postgraduate	Yes	Urban	No	No	Yes	No	No	I don't know		Not infected	3	Yes	Agree	Agree	Agree	Agree	Agree	Agree	Agree	No		Yes	Yes	Yes	Yes	3	4	Unwilling to receive 3rd dose (Please proceed to question 22a)	I don't think it's necessary as the number of COVID-19 cases has gone down already	5	4	It gives me a sense of security knowing that the patient is not infected					
Timestamp	senthilvasansuwarna@gmail.com	Agree	18-30 years old	Female	Indian	Single	Healthy adults	Undergraduate	Yes	Urban	No	Yes	Yes	No	I don't know	I don't know		Yes and confirmed	4	Yes	Agree	Agree	Agree	Agree	Neutral	Agree	Disagree	Yes	Vaccines are dangerous/harmful and can cause serious side effects	Yes	Yes	No	Yes	3	3	Taken only 1st, 2nd, and 3rd dose but willing to receive 4th dose		4	4	If the patient is tested positive, I can plan an alternate treatment option					
Timestamp	tina@pidc.edu.my	Agree	31-40 years old	Female	Indian	Married	Children	Postgraduate	Yes	Urban	No	No	Yes	No	No	I don't know		I don't know	3	No	Neutral	Neutral	Neutral	Agree	Strongly agree	Strongly agree	Strongly agree	No		Yes	No	No	No	5	1	Received 1st, 2nd, and 3rd dose	I don't think it's necessary as the number of COVID-19 cases has gone down already	4	1						
Timestamp	jalison@pidc.edu.my	Agree	31-40 years old	Male	Indian	Married	Non-applicable	Postgraduate	Yes	Urban	No	Yes	Yes	No	No	I don't know		I don't know	1	Yes	Neutral	Neutral	Neutral	Neutral	Disagree	Disagree	Neutral	No		Yes	No	No	No	4	4	Received 1st, 2nd, and 3rd dose	I'm worried about the side effect of the booster shots	1	3						
Timestamp	ykyogais08@gmail.com	Agree	41-50 years old	Male	Indian	Married	Healthy adults	Postgraduate	No	Urban	No	No	Yes	Yes	Yes	Yes and confirmed	2 weeks	Yes and confirmed	4	Yes	Strongly agree	Strongly agree	Strongly agree	Strongly agree	Strongly agree	Strongly agree	Strongly disagree	No		Yes	Yes	Yes	Yes	1	1	Received 1st, 2nd, 3rd, and 4th dose		1							
Timestamp	aparnnadayanidhi@gmail.com	Agree	31-40 years old	Female	Indian	Married	Children	Postgraduate	Yes	Rural	No	No	No		No	Not infected		Not infected	3	No	Neutral	Agree	Agree	Neutral	Neutral	Agree	Disagree	Yes	Vaccines are dangerous/harmful and can cause serious side effects	Yes	Yes	Yes	Yes	3	4	Received 1st, 2nd and 3rd dose	I'm worried about the side effect of the booster shots	4	4	If the patient is tested positive, I can plan an alternate treatment option					
Timestamp	sharma.ganesha@gmail.com	Agree	18-30 years old	Male	Indian	Single	Individuals with chronic diseases	Undergraduate	No	Urban	No	Yes	Yes	No	Yes	I don't know		Yes and confirmed	3	Yes	Agree	Strongly agree	Agree	Agree	Agree	Neutral	Strongly disagree	Yes	Vaccines are used for government control	Yes	Yes	Yes	Yes	1	1	Received 1st, 2nd, and 3rd dose		4							
Timestamp	shalininair1328@gmail.com	Agree	18-30 years old	Female	Indian	Single	Individuals with chronic diseases	Undergraduate	Yes	Urban	No	No	Yes	Yes	Yes	Not infected		Yes and confirmed	5	Yes	Strongly agree	Agree	Strongly agree	Agree	Strongly agree	Agree	Strongly disagree	Yes	Vaccines are dangerous/harmful and can cause serious side effects	Yes	Yes	Yes	Yes	3	4	Received 1st, 2nd, and 3rd dose	I'm worried about the side effects of the booster shots	2	3						
Timestamp	dnessha@gmail.com	Agree	18-30 years old	Female	Indian	Single	Healthy adults	Undergraduate	Yes	Urban	No	Yes	No		No	Not infected		Yes and confirmed	3	No	Neutral	Agree	Agree	Agree	Neutral	Agree	Neutral	Yes	Vaccines are dangerous/harmful and can cause serious side effects	Yes	Yes	Yes	Yes	3	2	Received 1st, 2nd, and 3rd dose		4	3						
Timestamp	janjan.cheryl@gmail.com	Agree	18-30 years old	Female	Indian	Single	Individuals with chronic diseases	Undergraduate	Yes	Urban	No	Yes	No	No	No	Not infected		Not infected	4	No	Neutral	Neutral	Neutral	Agree	Agree	Agree	Strongly disagree	Yes	Vaccines are used for government control	Yes	Yes	Yes	Yes	3	3	Received 1st, 2nd, and 3rd dose	I don't think it's necessary as the number of COVID-19 cases has gone down already	5	5	Helps us monitor the current rate of infection among the patients					
Timestamp	jasini.k@gmail.com	Agree	18-30 years old	Female	Indian	Single	Individuals older than 65 years	Postgraduate	No	Urban	No	Yes	Yes	No	Yes	Yes and confirmed	1 week	I don't know	3	Yes	Agree	Strongly agree	Strongly agree	Strongly agree	Agree	Agree	Neutral	Yes	I've heard all of the above	Yes	Yes	Yes	Yes	2	1	Received 1st, 2nd, and 3rd dose		4							
Timestamp	archanaasivadas@gmail.com	Agree	18-30 years old	Female	Indian	Single	Individuals older than 65 years	Undergraduate	Yes	Urban	No	Yes	Yes	No	Yes	Not infected		I don't know	2	Yes	Strongly agree	Strongly agree	Strongly agree	Strongly agree	Strongly agree	Strongly agree	Disagree	Yes	Vaccines are dangerous/harmful and can cause serious side effects	Yes	Yes	Yes	Yes	1	4	Received 1st, 2nd, and 3rd dose	I feel that the first and second doses are already sufficient to keep me protected from the virus	5	5	If the patient is tested positive, I can plan an alternate treatment option					
Timestamp	anfi892001@gmail.com	Agree	18-30 years old	Female	Bumiputera	Single	Healthy adults	Undergraduate	Yes	Urban	No	Yes	Maybe	No	Yes	Not infected		Not infected	3	Yes	Neutral	Agree	Strongly agree	Strongly agree	Strongly agree	Strongly agree	Neutral	Yes	Vaccines are dangerous/harmful and can cause serious side effects	Yes	Yes	Yes	Yes	3	1	Taken only 1st, 2nd, and 3rd dose but willing to receive 4th dose		5	5	If the patient is tested positive, I can plan an alternate treatment option					
Timestamp	kirtiga1511@gmail.com	Agree	18-30 years old	Female	Indian	Single	Healthy adults	Undergraduate	Yes	Urban	No	No	Yes	Yes	Yes	Yes and confirmed	1 week	Yes and confirmed	4	Yes	Strongly agree	Agree	Strongly agree	Strongly agree	Strongly agree	Strongly agree	Strongly agree	Yes	Vaccines are unnecessary as our body can fight off diseases on its own	Yes	Yes	Yes	No	3	1	Received 1st and 2nd dose	I don't think it's necessary as the number of COVID-19 cases has gone down already	5	5	If the patient is tested positive, I can plan an alternate treatment option					
Timestamp	manmeetbariar@gmail.com	Agree	18-30 years old	Female	Punjabi	Single	Healthy adults	Undergraduate	Yes	Urban	Yes	Yes	Yes	No	Yes	Yes and confirmed	Can't remember, somewhere mid 2023, lasted about 4 days	Yes and confirmed	3	Yes	Strongly agree	Agree	Strongly agree	Agree	Strongly agree	Agree	Agree	Yes	Vaccines are dangerous/harmful and can cause serious side effects	Yes	Yes	Yes	Yes	1	1	Unwilling to receive 4th dose (Please proceed to question 22a)	New side effects and many information hidden have recently come out which worries me	5	4	It gives me a sense of security knowing that the patient is not infected					
Timestamp	kelvinhowh@gmail.com	Agree	18-30 years old	Male	Chinese	Single	Healthy adults	Undergraduate	Yes	Urban	Yes	Yes	Yes	Yes	No	I don't know		Yes and confirmed	3	Yes	Agree	Agree	Agree	Agree	Agree	Agree	Disagree	Yes	Vaccines are dangerous/harmful and can cause serious side effects	Yes	Yes	Yes	Yes	3	3	Received 1st, 2nd, and 3rd dose		5	4	If the patient is tested positive, I can plan an alternate treatment option					
Timestamp	janitra1@gmail.com	Agree	18-30 years old	Female	Indian	Single	Healthy adults	Undergraduate	No	Urban	No	Yes	Yes	No	Yes	Yes and confirmed	1 week	Yes and confirmed	3	Yes	Disagree	Agree	Agree	Agree	Strongly agree	Strongly agree	Strongly agree	Yes	Vaccines are dangerous/harmful and can cause serious side effects	Yes	Yes	Yes	Yes	3	2	Received 1st, 2nd, and 3rd dose	I feel that the first and second doses are already sufficient to keep me protected from the virus	5							
Timestamp	selva030867@gmail.com	Agree	51-60 years old	Male	Indian	Married	Healthy adults	Postgraduate	No	Urban	No	No	Yes	Yes	No	Not infected		Yes and confirmed	3	Yes	Agree	Agree	Strongly agree	Agree	Strongly agree	Strongly agree	Neutral	Yes	Vaccines are dangerous/harmful and can cause serious side effects	Yes	Yes	Yes	Yes	1	1	Received 1st, 2nd, and 3rd dose	I feel that the first and second doses are already sufficient to keep me protected from the virus	5							
Timestamp	ash@avinityanalytics.com	Agree	18-30 years old	Female	Indian	Single	Non-applicable	Undergraduate	No	Urban	No	No	Yes	No	I don't know	Not infected		Yes and confirmed	2	No	Neutral	Neutral	Neutral	Neutral	Neutral	Neutral	Neutral	No		Yes	Yes	Yes	Yes	3	3	Received 1st, 2nd, and 3rd dose		3							
Timestamp	kashvyin@gmail.com	Agree	18-30 years old	Male	Indian	Single	Non-applicable	Undergraduate	No	Urban	No	No	Yes	No	No	I don't know		Not infected	2	No	Agree	Agree	Agree	Agree	Agree	Agree	Agree	Yes	Vaccines are dangerous/harmful and can cause serious side effects	Yes	Yes	Yes	Yes	3	4	Received 1st, 2nd and 3rd dose		5							
Timestamp	saravanan@kowabunga.global	Agree	41-50 years old	Male	Indian	Married	Children	Postgraduate	No	Urban	No	No	Yes	Yes	Yes	Yes and confirmed	2weeks	Yes and confirmed	4	Yes	Agree	Agree	Agree	Agree	Neutral	Agree	Agree	Yes	Vaccines are dangerous/harmful and can cause serious side effects	Yes	Yes	No	Yes	4	3	Received 1st and 2nd dose	I'm worried about the side effects of the booster shots	4	2	It gives me a sense of security knowing that the patient is not infected					
Timestamp	fatinizulaikha17@gmail.com	Agree	18-30 years old	Female	Malay	Single	Healthy adults	Undergraduate	Yes	Urban	No	No	Maybe	No	I don't know	Not infected		Yes and confirmed	4	Yes	Agree	Agree	Agree	Agree	Agree	Neutral	Neutral	Yes	Vaccines are dangerous/harmful and can cause serious side effects	Yes	Yes	Yes	Yes	4	4	Received 1st, 2nd, 3rd, and 4th dose		5	5	If the patient is tested positive, I can plan an alternate treatment option					
Timestamp	drfawazsiddiqui@gmail.com	Agree	41-50 years old	Male	Indian	Married	Healthy adults	Postgraduate	Yes	Urban	No	No	Yes	No	No	I don't know		I don't know	3	Yes	Strongly disagree	Agree	Neutral	Agree	Neutral	Neutral	Strongly disagree	No		Yes	Yes	Yes	Yes	2	1	Unwilling to receive 3rd dose (Please proceed to question 22a)	I'm worried about the side effects of the booster shots	4	1						
Timestamp	kumariupsi@gmail.com	Agree	41-50 years old	Female	Indian	Married	Children	Postgraduate	No	Urban	No	No	Yes	No	No	Not infected		Not infected	3	Yes	Neutral	Agree	Strongly agree	Agree	Strongly agree	Agree	Neutral	No		Yes	Yes	Yes	Yes	3	2	Received 1st and 2nd dose	I feel that the first and second doses are already sufficient to keep me protected from the virus	4							
Timestamp	francispltan@gmail.com	Agree	51-60 years old	Male	Chinese	Divorced/separated/widowed	Children	Undergraduate	Yes	Urban	Yes	No	No	No	Yes	Yes and confirmed	5 days	I don't know	3	Yes	Strongly disagree	Strongly agree	Strongly agree	Strongly agree	Strongly agree	Strongly agree	Strongly agree	Yes	Vaccines are dangerous/harmful and can cause serious side effects	Yes	Yes	Yes	Yes	1	5	Received 1st, 2nd, and 3rd dose	I feel that the first and second doses are already sufficient to keep me protected from the virus	5	5	It gives me a sense of security knowing that the patient is not infected					
Timestamp	roshinisara09@gmail.com	Agree	18-30 years old	Female	Indian	Single	Healthy adults	Diploma	Yes	Urban	No	Yes	Yes	Yes	No	Not infected		Not infected	3	Yes	Agree	Agree	Agree	Agree	Agree	Agree	Neutral	No		Yes	Yes	Yes	Yes	3	3	Received 1st, 2nd, and 3rd dose		3	4	It gives me a sense of security knowing that the patient is not infected					
Timestamp	bhisshmended@gmail.com	Agree	18-30 years old	Male	Indian	Single	Healthy adults	Undergraduate	No	Urban	No	No	Yes	No	No	Not infected		Not infected	3	No	Strongly disagree	Strongly disagree	Strongly disagree	Neutral	Disagree	Disagree	Neutral	Yes	Vaccines are dangerous/harmful and can cause serious side effects	Yes	Yes	Yes	Yes	5	2	Received 1st and 2nd dose	I feel that the first and second doses are already sufficient to keep me protected from the virus	4							
Timestamp	bmunchen1979@gmail.com	Agree	41-50 years old	Male	Indian	Married	Children	Postgraduate	No	Urban	No	No	Yes	No	Yes	Yes and confirmed	1 week	Yes and confirmed	4	No	Agree	Strongly agree	Strongly agree	Strongly agree	Strongly agree	Strongly agree	Neutral	No		Yes	Yes	Yes	Yes	4	5	Taken only 1st and 2nd dose but willing to receive 3rd and 4th dose	I feel that the first and second doses are already sufficient to keep me protected from the virus	5							
Timestamp	nantha.p1235@gmail.com	Agree	51-60 years old	Male	Indian	Married	Children	Diploma	Yes	Rural	No	No	Yes	Yes	Yes	I don't know		I don't know	2	Yes	Neutral	Neutral	Neutral	Neutral	Neutral	Neutral	Neutral	No	Vaccines are ineffective	Yes	Yes	Yes	Yes	2	3	Received 1st and 2nd dose	I don't think it's necessary as the number of COVID-19 cases has gone down already	2	2	Helps us monitor the current rate of infection among the patients					
Timestamp	kasturiraaj2306@gmail.com	Agree	31-40 years old	Female	Indian	Married	Healthy adults	Undergraduate	Yes	Urban	No	No	Yes	No	Yes	Not infected		Not infected	4	Yes	Agree	Strongly agree	Agree	Neutral	Strongly agree	Agree	Disagree	No		Yes	Yes	Yes	Yes	2	4	Received 1st and 2nd dose	I don't think it's necessary as the number of COVID-19 cases has gone down already	5	4	If the patient is tested positive, I can plan an alternate treatment option					
Timestamp	shantiselva25@gmail.com	Agree	51-60 years old	Female	Indian	Married	Healthy adults	Diploma	No	Urban	No	Yes	Yes	No	No	Not infected		Not infected	3	No	Disagree	Neutral	Neutral	Neutral	Neutral	Disagree	Strongly disagree	No		Yes	Yes	Yes	Yes	1	1	Received 1st, 2nd, and 3rd dose		5							
Timestamp	jsvasan8600@gmail.com	Agree	51-60 years old	Male	Indian	Married	Children	Diploma	Yes	Urban	Yes	Yes	Yes	Yes	Yes	Yes and confirmed	2 - 3 wks	Yes and confirmed	4	Yes	Neutral	Strongly agree	Strongly agree	Agree	Agree	Agree	Agree	Yes	Side effects	Yes	Yes	Yes	Yes	3	5	Received 1st, 2nd, and 3rd dose	I feel that the first and second doses are already sufficient to keep me protected from the virus	3							
Timestamp	swathyraj.saras@gmail.com	Agree	41-50 years old	Female	Indian	Married	Individuals older than 65 years	Postgraduate	No	Urban	No	No	Yes	No	No	Not infected		Not infected	3	Yes	Disagree	Agree	Disagree	Agree	Agree	Neutral	Strongly disagree	Yes	Vaccines are unnecessary as our body can fight off diseases on its own	Yes	No	Yes	Yes	3	3	Unwilling to receive 4th dose (Please proceed to question 22a)	I'm worried about the side effects of the booster shots	3							
Timestamp	vaanishreekumar98@gmail.com	Agree	18-30 years old	Female	Indian	Single	Healthy adults	Undergraduate	Yes	Urban	No	No	Yes	No	Yes	Yes and confirmed	1 week	Yes and confirmed	5	Yes	Strongly agree	Strongly agree	Strongly agree	Strongly agree	Strongly agree	Strongly agree	Strongly disagree	Yes	Vaccines are dangerous/harmful and can cause serious side effects	No	Yes	No	No	1	1	Received 1st and 2nd dose	I feel that the first and second doses are already sufficient to keep me protected from the virus	5	5	If the patient is tested positive, I can plan an alternate treatment option					
Timestamp	jaydcn062@gmail.com	Agree	41-50 years old	Male	Indian	Married	Children	Postgraduate	No	Urban	No	No	Yes	No	Yes	Not infected		Not infected	3	Yes	Strongly agree	Strongly agree	Agree	Strongly agree	Neutral	Neutral	Strongly disagree	No		Yes	Yes	Yes	Yes	1	3	Received 1st and 2nd dose	I feel that the first and second doses are already sufficient to keep me protected from the virus	1							
Timestamp	pirdinas1@gmail.com	Agree	18-30 years old	Female	Malay	Single	Healthy adults	Undergraduate	Yes	Urban	No	No	No	No	Yes	I don't know		I don't know	1	No	Agree	Strongly agree	Strongly agree	Strongly agree	Strongly agree	Strongly agree	Neutral	No		Yes	Yes	Yes	Yes	1	3	Taken only 1st and 2nd dose but willing to receive 3rd and 4th dose		5	3						
Timestamp	syazellah@gmail.com	Agree	41-50 years old	Female	Malay	Married	Children	Undergraduate	Yes	Urban	No	Yes	Yes	No	Yes	I don't know		I don't know	4	Yes	Agree	Agree	Agree	Agree	Agree	Agree	Disagree	No		Yes	Yes	Yes	Yes	2	3	Received 1st, 2nd, and 3rd dose		5	2						
Timestamp	dpalanisamy03@gmail.com	Agree	51-60 years old	Male	Indian	Married	Non-applicable	Diploma	Yes	Urban	Yes	No	Yes	No	Yes	Not infected		I don't know	2	Yes	Disagree	Disagree	Disagree	Disagree	Disagree	Disagree	Neutral	Yes	Vaccines are dangerous/harmful and can cause serious side effects	No	No	No	No	2	4	Received 1st, 2nd, and 3rd dose	I feel that the first and second doses are already sufficient to keep me protected from the virus	5	5	Helps us monitor the current rate of infection among the patients					
Timestamp	shohinni.vijaykumar@gmail.com	Agree	18-30 years old	Female	Indian	Single	Healthy adults	Undergraduate	Yes	Urban	No	Yes	Yes	Yes	No	Not infected		Not infected	4	Yes	Neutral	Strongly agree	Strongly agree	Strongly agree	Strongly agree	Strongly agree	Neutral	No		Yes	Yes	Yes	Yes	3	3	Taken only 1st, 2nd, and 3rd dose but willing to receive 4th dose		4	3						
Timestamp	kandy.1962@gmail.com	Agree	31-40 years old	Female	Indian	Married	Healthy adults	Postgraduate	No	Urban	Yes	Yes	Yes	No	Yes	Not infected		Not infected	5	Yes	Disagree	Disagree	Disagree	Disagree	Disagree	Disagree	Disagree	Yes	Vaccines are unnecessary as our body can fight off diseases on its own	No	No	No	No	4	4	Received 1st, 2nd, and 3rd dose	I'm worried about the side effects of the booster shots	1	4	If the patient is tested positive, I can plan an alternate treatment option					
Timestamp	naylyputri07@gmail.com	Agree	18-30 years old	Female	Malay	Single	Healthy adults	Undergraduate	Yes	Urban	No	No	Yes	No	No	I don't know		I don't know	4	No	Agree	Agree	Agree	Agree	Neutral	Agree	Neutral	No		Yes	Yes	Yes	Yes	1	1	Taken only 1st, 2nd, and 3rd dose but willing to receive 4th dose		5	4	It gives me a sense of security knowing that the patient is not infected					
Timestamp	dharenrow22@gmail.com	Agree	18-30 years old	Male	Indian	Single	Individuals with chronic diseases	Undergraduate	No	Urban	No	No	No		I don't know	I don't know		I don't know	3	No	Strongly disagree	Strongly disagree	Neutral	Strongly agree	Neutral	Neutral	Strongly disagree	Yes	Vaccines are dangerous/harmful and can cause serious side effects	No	Yes	No	No	2	2	Unwilling to receive 3rd dose (Please proceed to question 22a)	I'm worried about the side effects of the booster shots	2							
Timestamp	euyeanchan@gmail.com	Agree	18-30 years old	Female	Chinese	Single	Individuals older than 65 years	Postgraduate	Yes	Urban	No	Yes	Yes	Yes	Yes	Not infected		Not infected	3	Yes	Agree	Agree	Agree	Agree	Neutral	Agree	Disagree	Yes	Vaccines are dangerous/harmful and can cause serious side effects	Yes	Yes	Yes	Yes	3	4	Taken only 1st and 2nd dose but willing to receive 3rd dose	I'm worried about the side effects of the booster shots	3	4	If the patient is tested positive, I can plan an alternate treatment option					
Timestamp	prislyakanka@gmail.com	Agree	18-30 years old	Female	Indian	Single	Healthy adults	Undergraduate	Yes	Urban	No	No	Maybe		No	I don't know		I don't know	3	No	Neutral	Strongly agree	Agree	Strongly agree	Neutral	Agree	Disagree	Yes	Vaccines are dangerous/harmful and can cause serious side effects	Yes	Yes	Yes	Yes	2	4	Received 1st, 2nd, and 3rd dose		5	4	Helps us monitor the current rate of infection among the patients					
Timestamp	nivhashinirameshdr@gmail.com	Agree	18-30 years old	Female	Indian	Single	Healthy adults	Undergraduate	Yes	Urban	No	No	Yes	Yes	Yes	Not infected		Not infected	1	Yes	Neutral	Agree	Agree	Neutral	Strongly agree	Strongly agree	Disagree	Yes	Vaccines are dangerous/harmful and can cause serious side effects	Yes	Yes	Yes	Yes	4	4	Unwilling to receive 3rd dose (Please proceed to question 22a)	I'm worried about the side effects of the booster shots	4	3	If the patient is tested positive, I can plan an alternate treatment option					
Timestamp	arulanandammartin@gmail.com	Agree	71-80 years old	Male	Indian	Married	Individuals older than 65 years	Undergraduate	Yes	Urban	Yes	Yes	No		I don't know	Yes and confirmed	3 to4 days	I don't know	4	No	Neutral	Agree	Agree	Agree	Agree	Agree	Agree	No		Yes	Yes	No	No	3	3	Received 1st and 2nd dose	I'm worried about the side effects of the booster shots	5	3						
Timestamp	bhaalaa.g@gmail.com	Agree	18-30 years old	Male	Indian	Single	Healthy adults	Undergraduate	Yes	Urban	No	No	Yes	Yes	Yes	Not infected		Not infected	4	Yes	Neutral	Strongly agree	Strongly agree	Strongly agree	Strongly agree	Strongly agree	Strongly agree	Yes	Vaccines are dangerous/harmful and can cause serious side effects	Yes	Yes	Yes	Yes	1	3	Received 1st, 2nd, and 3rd dose		4	5	If the patient is tested positive, I can plan an alternate treatment option					
Timestamp	yamunaa0707@gmail.com	Agree	18-30 years old	Female	Indian	Single	Healthy adults	Undergraduate	Yes	Rural	Yes	No	Yes	No	Yes	Not infected		Not infected	3	Yes	Strongly agree	Agree	Strongly agree	Strongly agree	Strongly agree	Strongly agree	Agree	No		Yes	Yes	Yes	Yes	1	1	Received 1st, 2nd, and 3rd dose		3	4						
Timestamp	felicitysln@gmail.com	Agree	31-40 years old	Female	bumiputra	Single	Healthy adults	Undergraduate	Yes	Urban	No	No	Yes	Yes	No	Yes and confirmed	around 3-4 days	I don't know	4	No	Disagree	Agree	Agree	Neutral	Neutral	Agree	Agree	Yes	Vaccines are dangerous/harmful and can cause serious side effects	Yes	Yes	Yes	Yes	2	3	Taken only 1st and 2nd dose but willing to receive 3rd dose		4	4	If the patient is tested positive, I can plan an alternate treatment option					
Timestamp	cgeet16@gmail.com	Agree	41-50 years old	Female	Indian	Single	Individuals older than 65 years	Postgraduate	No	Urban	No	No	Yes	No	Yes	Not infected		Not infected	3	No	Strongly disagree	Strongly disagree	Strongly disagree	Strongly disagree	Strongly disagree	Neutral	Disagree	No		Yes	Yes	Yes	Yes	4	3	Received 1st, 2nd, and 3rd dose	I didn't know	4	4	It gives me a sense of security knowing that the patient is not infected					
Timestamp	sashtakesphotos2023@gmail.com	Agree	18-30 years old	Female	Indian	Single	Non-applicable	Secondary education	No	Urban	No	No	Yes	No	Yes	I don't know		I don't know	1	No	Agree	Agree	Strongly agree	Strongly agree	Agree	Agree	Neutral	No		Yes	Yes	Yes	Yes	5	4	Received 1st and 2nd dose	The first and second dose was organised by my school so I did not have to go out of my way to get the 3rd or booster shots. For me I think I didn’t get the booster because it was going out of my way, and also I’m not a fan of needles. If the decision is made for me, I will go, but if I have to make the decision, it will be difficult for me to go out of my way and get the booster.	4		It lowers the risk of people getting the infection and also reduces the number of people who will get it due to it being spread.					
Timestamp	arumugamparimaladevi@gmail.com	Agree	61-70 years old	Female	Indian	Married	Healthy adults	Other	No	Urban	Yes	Yes	Yes	No	No	Not infected		I don't know	1	No	Disagree	Neutral	Neutral	Neutral	Neutral	Neutral	Neutral	No	Vaccines are unnecessary as our body can fight off diseases on its own	Yes	Yes	Yes	No	5	5	Received 1st, 2nd, and 3rd dose	I don't think it's necessary as the number of COVID-19 cases has gone down already	5		Helps us monitor the current rate of infection among the patients					
Timestamp	tinesh31@gmail.com	Agree	18-30 years old	Male	Indian	Single	Healthy adults	Undergraduate	No	Urban	No	No	Yes	No	No	Not infected		Yes and confirmed	2	No	Neutral	Agree	Agree	Agree	Neutral	Neutral	Neutral	Yes	Vaccines are dangerous/harmful and can cause serious side effects	Yes	Yes	Yes	Yes	3	3	Unwilling to receive 3rd dose (Please proceed to question 22a)	I feel that the first and second doses are already sufficient to keep me protected from the virus	3	3	It gives me a sense of security knowing that the patient is not infected					
Timestamp	malan8920@gmail.com	Agree	31-40 years old	Male	Indian	Married	Healthy adults	Postgraduate	No	Urban	No	No	Yes	No	I don't know	Not infected		Not infected	3	Yes	Neutral	Neutral	Neutral	Neutral	Neutral	Neutral	Neutral	No		Yes	Yes	Yes	Yes	1	1	Received 1st, 2nd, and 3rd dose	Didn’t make any attempt for the fourth to be exact	5							
Timestamp	mafiqwira@gmail.com	Agree	18-30 years old	Male	Malay	Single	Healthy adults	Undergraduate	Yes	Urban	No	No	Yes	No	No	I don't know		Not infected	3	No	Agree	Agree	Agree	Agree	Agree	Agree	Neutral	Yes	Vaccines are dangerous/harmful and can cause serious side effects	Yes	Yes	Yes	Yes	4	3	Taken only 1st and 2nd dose but willing to receive 3rd and 4th dose	I don't think it's necessary as the number of COVID-19 cases has gone down already	5	5	It gives me a sense of security knowing that the patient is not infected					
Timestamp	renishavikneshwary@gmail.com	Agree	18-30 years old	Female	Indian	Single	Healthy adults	Undergraduate	Yes	Urban	No	No	Yes	No	No	Not infected		Not infected	4	Yes	Neutral	Agree	Neutral	Agree	Agree	Neutral	Disagree	No		Yes	Yes	Yes	No	3	3	Received 1st, 2nd, and 3rd dose	I'm worried about the side effects of the booster shots	3	4	If the patient is tested positive, I can plan an alternate treatment option					
Timestamp	elina.sheryn.victor@gmail.com	Agree	18-30 years old	Female	Indian	Single	Individuals with chronic diseases	Undergraduate	Yes	Urban	No	Yes	Yes	No	Yes	Not infected		Not infected	3	Yes	Agree	Agree	Agree	Agree	Agree	Agree	Strongly disagree	Yes	Vaccines are dangerous/harmful and can cause serious side effects	Yes	Yes	Yes	Yes	2	3	Unwilling to receive 3rd dose (Please proceed to question 22a)	Advised by my doctor not to proceed with the third dose due to my health complications	3	4	If the patient is tested positive, I can plan an alternate treatment option					
Timestamp	srienynadia@gmail.com	Agree	18-30 years old	Female	Malay	Single	Healthy adults	Undergraduate	Yes	Urban	No	No	Maybe		I don't know	I don't know		I don't know	3	No	Strongly agree	Strongly agree	Strongly agree	Strongly agree	Strongly agree	Strongly agree	Neutral	No		Yes	Yes	Yes	Yes	4	3	Received 1st, 2nd, and 3rd dose		5	4	Prevent from spreading to patients and healthcare workers					
Timestamp	claireleelanigomez@gmail.com	Agree	18-30 years old	Female	Indian	Single	Non-applicable	Undergraduate	No	Urban	No	Yes	Yes	Yes	I don't know	I don't know		I don't know	3	No	Agree	Agree	Strongly agree	Strongly disagree	Neutral	Agree	Strongly disagree	No		Yes	Yes	Yes	Yes	3	3	Received 1st and 2nd dose	I feel that the first and second doses are already sufficient to keep me protected from the virus	5							
Timestamp	pathmasaravanan@gmail.com	Agree	41-50 years old	Male	Indian	Married	Non-applicable	Postgraduate	No	Urban	No	No	Yes	Yes	Yes	Yes and confirmed	2 weeks	Yes and confirmed	3	Yes	Agree	Agree	Agree	Agree	Agree	Agree	Agree	No	Vaccines are dangerous/harmful and can cause serious side effects	Yes	Yes	Yes	Yes	4	4	Received 1st, 2nd, and 3rd dose	I'm worried about the side effects of the booster shots	4	4	If the patient is tested positive, I can plan an alternate treatment option					
Timestamp	stacengsy@gmail.com	Agree	18-30 years old	Female	Chinese	Single	Non-applicable	Postgraduate	Yes	Rural	No	No	Yes	Yes	No	Not infected		Not infected	5	Yes	Disagree	Agree	Agree	Agree	Neutral	Neutral	Strongly disagree	Yes	experienced undesirable s/e	Yes	Yes	Yes	Yes	4	2	Received 1st, 2nd, 3rd, and 4th dose		4	4	patients immune system may be down atm, hence whether is it still appropriate to undergo certain dental procedures etc					
Timestamp	asuras66@gmail.com	Agree	51-60 years old	Male	Indian	Married	Non-applicable	Other	No	Urban	No	No	Yes	No	No	Not infected		Not infected	3	No	Strongly disagree	Strongly disagree	Strongly disagree	Strongly disagree	Strongly disagree	Neutral	Disagree	No	Vaccines are ineffective	Yes	No	No	No	4	3	Received 1st and 2nd dose	I'm worried about the side effects of the booster shots	3							
Timestamp	aarani9804@gmail.com	Agree	18-30 years old	Female	Indian	Single	Healthy adults	Undergraduate	Yes	Urban	No	No	Yes	Yes	Yes	Not infected		Not infected	4	Yes	Agree	Agree	Agree	Agree	Agree	Agree	Agree	Yes	Vaccines are dangerous/harmful and can cause serious side effects	Yes	Yes	Yes	Yes	3	2	Received 1st and 2nd dose	I'm worried about the side effects of the booster shots	3	4	It gives me a sense of security knowing that the patient is not infected					
Timestamp	myramunirah12@gmail.com	Agree	18-30 years old	Female	Malay	Single	Healthy adults	Undergraduate	Yes	Urban	No	No	Maybe	No	No	Not infected		Not infected	2	Yes	Disagree	Agree	Agree	Agree	Agree	Agree	Neutral	No		Yes	Yes	Yes	Yes	3	3	Received 1st, 2nd, and 3rd dose		4	3						
Timestamp	indralekasaravanan@gmail.com	Agree	18-30 years old	Female	Indian	Single	Healthy adults	Postgraduate	No	Urban	No	No	Yes	No	No	Yes and confirmed	5 days	I don't know	1	No	Neutral	Agree	Agree	Agree	Agree	Agree	Disagree	Yes	Vaccines are dangerous/harmful and can cause serious side effects	Yes	Yes	No	No	3	4	Received 1st and 2nd dose	I'm worried about the side effects of the booster shots	4							
Timestamp	puneethariya84@gmail.com	Agree	31-40 years old	Female	Indian	Married	Children	Diploma	Yes	Urban	No	No	Yes	Yes	Yes	Yes and confirmed	2 weeks	Yes and confirmed	5	Yes	Strongly agree	Strongly agree	Strongly agree	Strongly agree	Strongly agree	Strongly agree	Strongly agree	No		Yes	Yes	Yes	Yes	5	1	Received 1st, 2nd, 3rd, and 4th dose		4	5	Helps us monitor the current rate of infection among the patients					
Timestamp	laashman96@gmail.com	Agree	18-30 years old	Male	Indian	Single	Individuals with chronic diseases	Undergraduate	No	Urban	No	No	Yes	No	I don't know	I don't know		I don't know	1	Yes	Strongly agree	Strongly agree	Strongly agree	Strongly agree	Strongly agree	Strongly agree	Strongly agree	Yes	Vaccines are dangerous/harmful and can cause serious side effects	No	No	No	No	5	5	Received 1st and 2nd dose	I'm worried about the side effects of the booster shots	1							
Timestamp	thashweeta15@gmail.com	Agree	18-30 years old	Female	Indian	Single	Individuals with chronic diseases	Undergraduate	No	Urban	No	No	Yes	Yes	I don't know	Not infected		Not infected	2	No	Neutral	Agree	Agree	Agree	Neutral	Neutral	Strongly disagree	Yes	Vaccines are dangerous/harmful and can cause serious side effects	Yes	Yes	Yes	Yes	5	1	Received 1st and 2nd dose	I'm worried about the side effects of the booster shots	3							
Timestamp	mohanaruben12@gmail.com	Agree	18-30 years old	Male	Indian	Single	Non-applicable	Undergraduate	Yes	Urban	No	Yes	No	No	Yes	I don't know		I don't know	3	Yes	Neutral	Agree	Agree	Neutral	Strongly agree	Agree	Neutral	Yes		Yes	Yes	Yes	Yes	2	4	Received 1st, 2nd, and 3rd dose		3	3						
Timestamp	wanbadrina@gmail.com	Agree	18-30 years old	Female	Malay	Single	Individuals older than 65 years	Undergraduate	Yes	Urban	Yes	Yes	Yes	No	Yes	I don't know		I don't know	2	Yes	Neutral	Strongly agree	Strongly agree	Strongly agree	Strongly agree	Strongly agree	Neutral	Yes	Vaccines are dangerous/harmful and can cause serious side effects	Yes	Yes	Yes	Yes	1	2	Taken only 1st, 2nd, and 3rd dose but willing to receive 4th dose		5	5	If the patient is tested positive, I can plan an alternate treatment option					
Timestamp	theeran91@gmail.com	Agree	31-40 years old	Male	Indian	Married	Healthy adults	Postgraduate	Yes	Urban	No	No	Yes	Yes	No	Not infected		Not infected	4	Yes	Neutral	Neutral	Neutral	Neutral	Neutral	Neutral	Neutral	No	Vaccines are used for government control	Yes	Yes	No	No	3	3	Received 1st, 2nd, 3rd, and 4th dose	I have taken all 4 doses	3	3	If the patient is tested positive, I can plan an alternate treatment option					
Timestamp	summethaparimalam23@gmail.com	Agree	18-30 years old	Female	Indian	Single	Non-applicable	Undergraduate	No	Urban	No	Yes	Yes	Yes	No	I don't know		I don't know	1	Yes	Neutral	Agree	Agree	Agree	Neutral	Neutral	Agree	Yes	Vaccines are dangerous/harmful and can cause serious side effects	Yes	Yes	Yes	Yes	5	5	Received 1st, 2nd, and 3rd dose	I feel that the first and second doses are already sufficient to keep me protected from the virus	3							
Timestamp	pavidhanisaa12@gmail.com	Agree	18-30 years old	Female	Indian	Single	Healthy adults	Undergraduate	Yes	Urban	No	Yes	Yes	Yes	I don't know	Not infected	-	Not infected	2	Yes	Neutral	Agree	Agree	Agree	Agree	Neutral	Neutral	Yes	Vaccines are dangerous/harmful and can cause serious side effects	Yes	Yes	No	No	3	4	Unwilling to receive 3rd dose (Please proceed to question 22a)	I feel that the first and second doses are already sufficient to keep me protected from the virus	3	4	It gives me a sense of security knowing that the patient is not infected					
Timestamp	pmdds19106026@mahsastudent.edu.my	Agree	18-30 years old	Female	Indian	Single	Healthy adults	Undergraduate	Yes	Urban	No	No	Yes	No	Yes	Yes and confirmed	1 week	Yes and confirmed	3	Yes	Agree	Agree	Agree	Agree	Neutral	Neutral	Disagree	Yes	Vaccines are used for government control	Yes	Yes	Yes	No	3	4	Received 1st, 2nd, and 3rd dose	I'm worried about the side effects of the booster shots	4	4	It gives me a sense of security knowing that the patient is not infected					
Timestamp	sangetanamuralie08@gmail.com	Agree	18-30 years old	Female	Indian	Single	Healthy adults	Undergraduate	Yes	Urban	No	No	Yes	No	No	Not infected		Not infected	3	Yes	Neutral	Agree	Agree	Agree	Agree	Neutral	Neutral	No		Yes	Yes	Yes	Yes	3	1	Received 1st and 2nd dose	I'm worried about the side effects of the booster shots	3	4	If the patient is tested positive, I can plan an alternate treatment option					
Timestamp	mekala.m1999@gmail.com	Agree	18-30 years old	Female	Indian	Single	Healthy adults	Undergraduate	Yes	Rural	No	No	Yes		I don't know	Not infected		I don't know	1	No	Agree	Strongly agree	Agree	Strongly agree	Strongly agree	Agree	Strongly disagree	No		Yes	Yes	Yes	Yes	3	3	Received 1st, 2nd, and 3rd dose		5	3						
Timestamp	raneshgk@gmail.com	Agree	51-60 years old	Female	Indian	Divorced/separated/widowed	Non-applicable	Postgraduate	No	Urban	Yes	Yes	Yes	Yes	No	I don't know		Not infected	3	Yes	Neutral	Strongly agree	Strongly agree	Strongly agree	Agree	Agree	Neutral	Yes	Vaccines are unnecessary as our body can fight off diseases on its own	Yes	Yes	Yes	Yes	1	1	Unwilling to receive 4th dose (Please proceed to question 22a)	I'm worried about the side effects of the booster shots	2							
Timestamp	muhammadizharismail@gmail.com	Agree	18-30 years old	Male	Malay	Single	Healthy adults	Undergraduate	Yes	Urban	No	Yes	No		No	Yes and confirmed	6 days	Yes and confirmed	4	No	Neutral	Strongly agree	Strongly agree	Strongly agree	Strongly agree	Strongly agree	Agree	Yes	Vaccines are dangerous/harmful and can cause serious side effects	Yes	Yes	Yes	Yes	5	5	Received 1st and 2nd dose		5	5	It gives me a sense of security knowing that the patient is not infected					
Timestamp	rekha1820@gmail.com	Agree	31-40 years old	Female	Indian	Married	Children	Undergraduate	No	Urban	No	No	Yes	No	No	Not infected		Not infected	4	Yes	Disagree	Agree	Agree	Neutral	Agree	Agree	Strongly disagree	No		Yes	Yes	Yes	Yes	3	1	Received 1st and 2nd dose	I don't think it's necessary as the number of COVID-19 cases has gone down already	4		Helps us monitor the current rate of infection among the patients					
Timestamp	uvarajan2020@gmail.com	Agree	18-30 years old	Male	Indian	Single	Individuals older than 65 years	Secondary education	No	Urban	No	No	Yes	Yes	No	Not infected		Not infected	4	Yes	Agree	Agree	Neutral	Strongly agree	Agree	Agree	Agree	No	Vaccines are used for government control	No	No	Yes	No	4	4	Received 1st, 2nd, and 3rd dose	I don't think it's necessary as the number of COVID-19 cases has gone down already	4	3	If the patient is tested positive, I can plan an alternate treatment option					
Timestamp	pmdds20106734@mahsastudent.edu.my	Agree	18-30 years old	Female	Indian	Single	Healthy adults	Undergraduate	Yes	Urban	No	Yes	Yes	No	Yes	Not infected		Not infected	4	Yes	Strongly agree	Strongly agree	Strongly agree	Strongly agree	Strongly agree	Strongly agree	Strongly disagree	Yes	Vaccines are used for government control	Yes	Yes	Yes	Yes	1	1	Taken only 1st, 2nd, and 3rd dose but willing to receive 4th dose		5	4	It gives me a sense of security knowing that the patient is not infected					
Timestamp	rekhavaradaraju@gmail.com	Agree	18-30 years old	Female	Indian	Single	Healthy adults	Undergraduate	Yes	Urban	No	No	Yes	Yes	Yes	Not infected		Not infected	3	Yes	Agree	Agree	Strongly agree	Neutral	Strongly agree	Strongly agree	Disagree	No		Yes	Yes	Yes	Yes	3	1	Received 1st, 2nd, and 3rd dose		4	5	Helps us monitor the current rate of infection among the patients					
Timestamp	choonchyeaw@gmail.com	Agree	61-70 years old	Male	Chinese	Divorced/separated/widowed	Children	Diploma	No	Urban	No	Yes	Yes	No	No	Not infected		Not infected	2	No	Strongly agree	Strongly agree	Strongly agree	Strongly agree	Strongly agree	Strongly agree	Strongly disagree	No		Yes	Yes	Yes	Yes	3	3	Received 1st, 2nd, and 3rd dose	I'm worried about the side effects of the booster shots	3							
Timestamp	leannelxe@gmail.com	Agree	18-30 years old	Female	Chinese	Single	Healthy adults	Undergraduate	Yes	Urban	No	No	Yes	No	I don't know	Not infected		Not infected	4	Yes	Agree	Strongly agree	Strongly agree	Agree	Neutral	Neutral	Neutral	Yes	Vaccines are dangerous/harmful and can cause serious side effects	Yes	Yes	Yes	Yes	5	1	Received 1st, 2nd, and 3rd dose		3	5	Helps us monitor the current rate of infection among the patients					
Timestamp	kasturiavalee@gmail.com	Agree	31-40 years old	Female	Indian	Married	Individuals with chronic diseases	Postgraduate	No	Urban	No	Yes	Yes	Yes	No	Yes and confirmed	3 days	Yes and confirmed	5	Yes	Strongly agree	Strongly agree	Strongly agree	Strongly agree	Strongly agree	Strongly agree	Strongly disagree	Yes	Vaccines are dangerous/harmful and can cause serious side effects	Yes	Yes	Yes	Yes	5	1	Received 1st, 2nd, and 3rd dose	I don't think it's necessary as the number of COVID-19 cases has gone down already	5							
Timestamp	vijayasurendran8@gmail.com	Agree	51-60 years old	Female	Indian	Married	Children	Undergraduate	Yes	Urban	Yes	Yes	Yes	Yes	Yes	Yes and confirmed	1 week	Yes and confirmed	4	Yes	Agree	Neutral	Agree	Agree	Neutral	Neutral	Neutral	Yes	Vaccines are dangerous/harmful and can cause serious side effects	Yes	Yes	No	Yes	5	5	Received 1st, 2nd, and 3rd dose	I don't think it's necessary as the number of COVID-19 cases has gone down already	4							
Timestamp	suveitra1@gmail.com	Agree	61-70 years old	Female	Chinese	Married	Individuals with chronic diseases	Undergraduate	No	Rural	Yes	No	No		No	Not infected		Yes and confirmed	2	No	Agree	Agree	Agree	Agree	Agree	Agree	Agree	No		Yes	Yes	Yes	Yes	3	3	Received 1st, 2nd, and 3rd dose	I feel that the first and second doses are already sufficient to keep me protected from the virus	4							
Timestamp	suvei10bal@gmail.com	Agree	31-40 years old	Male	Malay	Single	Healthy adults	Undergraduate	No	Urban	No	No	Yes	No	I don't know	I don't know		I don't know	3	Yes	Agree	Agree	Agree	Agree	Agree	Agree	Agree	No		Yes	Yes	Yes	Yes	3	3	Received 1st, 2nd, and 3rd dose		4							
Timestamp	pmdds19106077@mahsastudent.edu.my	Agree	18-30 years old	Male	Malay	Single	Healthy adults	Undergraduate	No	Urban	No	Yes	Yes	No	No	Not infected		Yes and confirmed	3	No	Agree	Agree	Agree	Agree	Agree	Agree	Agree	No		Yes	Yes	Yes	Yes	3	3	Received 1st, 2nd, and 3rd dose		4							
Timestamp	bmc954198@gmail.com	Agree	41-50 years old	Female	Indian	Married	Children	Postgraduate	No	Urban	No	No	Yes	No	No	I don't know		Yes and confirmed	4	Yes	Disagree	Disagree	Neutral	Disagree	Disagree	Neutral	Neutral	No		Yes	No	Yes	Yes	5	1	Received 1st, 2nd, and 3rd dose	I'm worried about the side effects of the booster shots	3							
Timestamp	lalithasivanganam@gmail.com	Agree	41-50 years old	Female	Indian	Married	Children	Postgraduate	No	Urban	No	Yes	Yes	Yes	No	Yes and confirmed		I don't know	1	No	Agree	Agree	Agree	Agree	Neutral	Neutral	Neutral	No	Vaccines are dangerous/harmful and can cause serious side effects	No	No	No	No	4	3	Received 1st, 2nd, and 3rd dose	I'm worried about the side effects of the booster shots	3							
Timestamp	seshmiithha@gmail.com	Agree	18-30 years old	Female	Indian	Single	Healthy adults	Undergraduate	No	Urban	No	Yes	Yes	No	Yes	Not infected		Not infected	3	No	Neutral	Agree	Strongly agree	Strongly agree	Strongly agree	Neutral	Agree	No		Yes	Yes	Yes	Yes	4	4	Received 1st, 2nd, and 3rd dose	I'm worried about the side effects of the booster shots	4							
Timestamp	ramesh5707@gmail.com	Agree	41-50 years old	Male	Indian	Married	Individuals older than 65 years	Other	No	Urban	Yes	Yes	Yes	Yes	Yes	I don't know		I don't know	2	Yes	Neutral	Agree	Neutral	Neutral	Neutral	Agree	Neutral	Yes	Vaccines are dangerous/harmful and can cause serious side effects	Yes	Yes	No	Yes	2	5	Received 1st and 2nd dose	I feel that the first and second doses are already sufficient to keep me protected from the virus	5	4	Helps us monitor the current rate of infection among the patients					
Timestamp	arunkumarsiva@gmail.com	Agree	31-40 years old	Male	Indian	Married	Children	Postgraduate	No	Urban	No	No	Yes	Yes	Yes	Yes and confirmed	3 weeks	I don't know	4	Yes	Neutral	Agree	Agree	Agree	Agree	Agree	Neutral	Yes	Vaccines are ineffective	Yes	Yes	Yes	Yes	2	3	Received 1st, 2nd, 3rd, and 4th dose		3	3						
Timestamp	hemendiran93@gmail.com	Agree	18-30 years old	Male	Indian	Married	Healthy adults	Undergraduate	Yes	Urban	No	No	Yes	No	Yes	Yes and confirmed	7 days	Yes and confirmed	3	Yes	Neutral	Strongly agree	Strongly agree	Strongly agree	Agree	Neutral	Disagree	Yes	Vaccines are used for government control	Yes	Yes	Yes	Yes	1	3	Received 1st, 2nd, and 3rd dose	I took the third dose	4	5	If the patient is tested positive, I can plan an alternate treatment option					
Timestamp	amasepusparani@gmail.com	Agree	41-50 years old	Female	Indian	Married	Healthy adults	Undergraduate	Yes	Urban	No	No	Yes	No	Yes	Not infected		Yes and confirmed	3	Yes	Agree	Agree	Agree	Agree	Agree	Agree	Neutral	Yes	Vaccines are ineffective	Yes	Yes	Yes	Yes	1	2	Taken only 1st, 2nd, and 3rd dose but willing to receive 4th dose		5							
Timestamp	dr.madhuniranjanswamy@gmail.com	Agree	31-40 years old	Male	Indian	Married	Individuals older than 65 years	Postgraduate	Yes	Urban	No	No	Yes	Yes	Yes	Yes and confirmed	1 week	Yes and confirmed	4	No	Agree	Agree	Agree	Agree	Agree	Neutral	Neutral	Yes	Both one and two	Yes	Yes	Yes	Yes	3	1	Received 1st, 2nd, 3rd, and 4th dose	I don't think it's necessary as the number of COVID-19 cases has gone down already	4	5	It gives me a sense of security knowing that the patient is not infected					
Timestamp	sanjaypragash87@gmail.com	Agree	18-30 years old	Male	Indian	Single	Individuals older than 65 years	Undergraduate	Yes	Urban	No	Yes	Yes	No	Yes	Not infected		Yes and confirmed	3	Yes	Strongly disagree	Strongly agree	Strongly agree	Neutral	Strongly agree	Strongly agree	Strongly agree	No		Yes	Yes	Yes	Yes	1	5	Received 1st, 2nd, and 3rd dose		5	2						
Timestamp	ziqinkee3357@gmail.com	Agree	18-30 years old	Female	Chinese	Single	Healthy adults	Undergraduate	Yes	Urban	No	Yes	Yes	No	No	Not infected		Not infected	4	No	Agree	Strongly agree	Strongly agree	Strongly agree	Strongly agree	Strongly agree	Strongly disagree	Yes	Vaccines are dangerous/harmful and can cause serious side effects	Yes	Yes	No	Yes	2	4	Received 1st, 2nd, and 3rd dose	I don't think it's necessary as the number of COVID-19 cases have gone down already	5	4	If the patient is tested positive, I can plan an alternate treatment option					
Timestamp	besreeeuen@gmail.com	Agree	18-30 years old	Male	Sino-dusun	Single	Individuals older than 65 years	Undergraduate	Yes	Urban	No	No	No		No	I don't know		I don't know	4	No	Strongly disagree	Neutral	Agree	Strongly agree	Strongly agree	Strongly agree	Neutral	Yes	Vaccines are dangerous/harmful and can cause serious side effects	Yes	Yes	Yes	Yes	4	3	Received 1st and 2nd dose	I have already been infected once, and I rely on natural immunity	4	4	If the patient is tested positive, I can plan an alternate treatment option					
Timestamp	pathmaranij@gmail.com	Agree	41-50 years old	Female	Indian	Married	Children	Diploma	No	Rural	No	No	Yes	Yes	Yes	Yes and confirmed	1 week	Yes and confirmed	5	Yes	Agree	Agree	Agree	Agree	Agree	Agree	Agree	Yes	Vaccines are dangerous/harmful and can cause serious side effects	No	No	No	No	3	3	Received 1st and 2nd dose	I feel that the first and second doses are already sufficient to keep me protected from the virus	3	3	Helps us monitor the current rate of infection among the patients					
Timestamp	shaatmi.19@gmail.com	Agree	18-30 years old	Female	Indian	Single	Healthy adults	Undergraduate	Yes	Urban	Yes	Yes	Yes	Yes	Yes	Not infected		Not infected	3	Yes	Neutral	Neutral	Neutral	Neutral	Neutral	Neutral	Neutral	Yes	Vaccines are dangerous/harmful and can cause serious side effects	Yes	Yes	Yes	Yes	1	1	Received 1st, 2nd, and 3rd dose	I don't think it's necessary as the number of COVID-19 cases have gone down already	3	5	Helps us monitor the current rate of infection among the patients					
Timestamp	jayaramanshankar70@gmail.com	Agree	51-60 years old	Male	Indian	Divorced/separated/widowed	Children	Undergraduate	No	Urban	No	Yes	Yes	Yes	Yes	Not infected		Not infected	3	No	Neutral	Strongly agree	Agree	Strongly agree	Strongly agree	Strongly agree	Strongly disagree	Yes	There are insufficient studies on the possible and/or serious side effects of vaccinations in general and transparency of disclosure to the public	No	Yes	No	No	5	1	Received 1st, 2nd, and 3rd dose	I'm worried about the side effects of the booster shots	3							
Timestamp	narenraven@gmail.com	Agree	18-30 years old	Male	Indian	Single	Healthy adults	Undergraduate	No	Urban	No	Yes	Yes	No	Yes	Yes and confirmed	5 days	Yes and confirmed	4	Yes	Agree	Strongly agree	Strongly agree	Strongly agree	Strongly agree	Strongly agree	Neutral	Yes	Vaccines are dangerous/harmful and can cause serious side effects	Yes	Yes	Yes	Yes	1	3	Taken only 1st, 2nd, and 3rd dose but willing to receive 4th dose		5							
Timestamp	luckymilk91@gmail.com	Agree	31-40 years old	Male	Chinese	Married	Healthy adults	Postgraduate	No	Urban	No	No	Yes	No	No	I don't know		I don't know	2	Yes	Agree	Agree	Agree	Agree	Agree	Agree	Agree	Yes	Vaccines are dangerous/harmful and can cause serious side effects	Yes	Yes	Yes	Yes	5	4	Received 1st and 2nd dose	I feel that the first and second doses are already sufficient to keep me protected from the virus	4							
Timestamp	andy14jo@gmail.com	Agree	18-30 years old	Female	Indian	Single	Healthy adults	Undergraduate	No	Urban	No	No	Yes	No	Yes	Not infected		Not infected	3	Yes	Strongly agree	Agree	Strongly agree	Strongly agree	Strongly agree	Agree	Strongly disagree	Yes	Vaccines are dangerous/harmful and can cause serious side effects	Yes	Yes	Yes	Yes	1	2	Received 1st, 2nd, and 3rd dose		4	4	Helps us monitor the current rate of infection among the patients					
Timestamp	divyanparam30@gmail.com	Agree	18-30 years old	Male	Indian	Single	Healthy adults	Undergraduate	No	Urban	No	Yes	Yes	Yes	No	Not infected		Not infected	3	No	Neutral	Neutral	Neutral	Neutral	Neutral	Neutral	Neutral	Yes	Vaccines are dangerous/harmful and can cause serious side effects	Yes	Yes	Yes	Yes	3	3	Received 1st, 2nd, and 3rd dose	I don't think it's necessary as the number of COVID-19 cases has gone down already	4	3	It gives me a sense of security knowing that the patient is not infected					
Timestamp	brennanbks25@gmail.com	Agree	18-30 years old	Male	Indian	Single	Healthy adults	Undergraduate	No	Urban	No	Yes	Yes	No	Yes	Yes and confirmed	1 week	Yes and confirmed	5	Yes	Neutral	Strongly agree	Strongly agree	Strongly agree	Strongly agree	Neutral	Neutral	No		Yes	Yes	Yes	Yes	1	2	Received 1st and 2nd dose	I feel that the first and second doses are already sufficient to keep me protected from the virus	1	5	It gives me a sense of security knowing that the patient is not infected					
Timestamp	laxmanparam@gmail.com	Agree	18-30 years old	Male	Indian	Single	Healthy adults	Undergraduate	No	Urban	No	Yes	Yes	Yes	Yes	Yes and confirmed	1 week	Yes and confirmed	3	No	Agree	Neutral	Neutral	Neutral	Agree	Agree	Agree	No		Yes	Yes	Yes	Yes	5	5	Received 1st, 2nd, 3rd and 4th dose		4							
Timestamp	amandaserenafoo@gmail.com	Agree	18-30 years old	Female	Chinese	Single	Healthy adults	Undergraduate	Yes	Urban	No	No	Yes	Yes	I don't know	I don't know		I don't know	3	No	Neutral	Strongly agree	Strongly agree	Agree	Neutral	Agree	Neutral	Yes	Vaccines are dangerous/harmful and can cause serious side effects	Yes	Yes	Yes	Yes	3	4	Taken only 1st and 2nd dose but willing to receive 3rd and 4th dose		3	4	It gives me a sense of security knowing that the patient is not infected					
Timestamp	audryyeulanda98@gmail.com	Agree	18-30 years old	Female	Sino-Kadazan	Single	Healthy adults	Undergraduate	Yes	Urban	No	No	Yes	Yes	Yes	Not infected	NRF	Not infected	4	Yes	Agree	Agree	Agree	Agree	Agree	Agree	Neutral	Yes	Vaccines are unnecessary as our body can fight off diseases on its own	Yes	Yes	Yes	Yes	5	4	Received 1st, 2nd, and 3rd dose	I feel that the first and second doses are already sufficient to keep me protected from the virus	4	5	If the patient is tested positive, I can plan an alternate treatment option					
Timestamp	vinayagar1235@gmail.com	Agree	41-50 years old	Female	Indian	Married	Individuals with chronic diseases	Postgraduate	No	Rural	No	No	Yes	Yes	No	Not infected		Not infected	4	Yes	Strongly agree	Neutral	Neutral	Neutral	Neutral	Neutral	Neutral	Yes	Vaccines are dangerous/harmful and can cause serious side effects	No	No	No	No	5	5	Received 1st, 2nd, and 3rd dose		3	5	If the patient is tested positive, I can plan an alternate treatment option					
Timestamp	aaronraj46@gmail.com	Agree	18-30 years old	Male	Indian	Single	Healthy adults	Undergraduate	No	Urban	No	No	Yes	Yes	Yes	Not infected		Not infected	3	No	Neutral	Neutral	Neutral	Neutral	Neutral	Neutral	Neutral	No		Yes	Yes	Yes	Yes	3	3	Received 1st, 2nd, and 3rd dose	I don't think it's necessary as the number of COVID-19 cases has gone down already	1	3	If the patient is tested positive, I can plan an alternate treatment option					
Timestamp	harnani.hasan0611@gmail.com	Agree	31-40 years old	Female	Malay	Married	Children	Postgraduate	Yes	Urban	No	No	Yes	No	No	Not infected		I don't know	3	Yes	Agree	Neutral	Neutral	Agree	Neutral	Neutral	Neutral	Yes	Vaccines are dangerous/harmful and can cause serious side effects	Yes	No	No	No	2	4	Received 1st, 2nd, and 3rd dose		4							
Timestamp	vankulagabu@gmail.com	Agree	18-30 years old	Female	Kadazan dusun	Single	Healthy adults	Undergraduate	No	Rural	No	Yes	Yes	No	Yes	Yes and confirmed	3 days	I don't know	3	No	Agree	Agree	Strongly agree	Strongly agree	Agree	Agree	Disagree	Yes	Vaccines are unnecessary as our body can fight off diseases on its own	Yes	Yes	Yes	Yes	2	2	Received 1st, 2nd, and 3rd dose	I don't think it's necessary as the number of COVID-19 cases has gone down already	3	4	If the patient is tested positive, I can plan an alternate treatment option					
Timestamp	aravind.tkv@gmail.com	Agree	31-40 years old	Male	Indian	Married	Children	Undergraduate	Yes	Urban	No	No	Yes	Yes	No	Not infected		I don't know	3	Yes	Strongly disagree	Neutral	Neutral	Neutral	Neutral	Neutral	Neutral	No		Yes	Yes	Yes	Yes	3	1	Unwilling to receive 3rd dose (Please proceed to question 22a)	I'm worried about the side effects of the booster shots	1							
Timestamp	maolo4884@gmail.com	Agree	31-40 years old	Male	Chinese	Married	Children	Postgraduate	Yes	Urban	No	No	Yes	No	Yes	I don't know		I don't know	3	Yes	Neutral	Neutral	Agree	Agree	Agree	Agree	Neutral	No		Yes	Yes	Yes	Yes	3	5	Received 1st, 2nd, and 3rd dose	I don't think it's necessary as the number of COVID-19 cases has gone down already	4	3						
Timestamp	mageswary.jayaram@gmail.com	Agree	31-40 years old	Female	Indian	Single	Healthy adults	Undergraduate	No	Urban	No	No	Yes	Yes	Yes	Not infected		Yes and confirmed	3	Yes	Agree	Neutral	Agree	Strongly agree	Strongly agree	Agree	Strongly disagree	No		Yes	Yes	Yes	Yes	1	1	Received 1st, 2nd, and 3rd dose	I'm worried about the side effects of the booster shots	3	3						
Timestamp	kal0106.ks@gmail.com	Agree	51-60 years old	Female	Indian	Married	Healthy adults	Diploma	Yes	Rural	No	No	Yes	No	Yes	Not infected		Yes and confirmed	5	Yes	Agree	Agree	Agree	Agree	Agree	Neutral	Disagree	No		Yes	Yes	Yes	Yes	3	4	Received 1st, 2nd, and 3rd dose	I'm worried about the side effects of the booster shots	3							
Timestamp	andreakneebone06@gmail.com	Agree	31-40 years old	Male	Chinese	Single	Healthy adults	Diploma	No	Urban	No	No	Maybe	No	I don't know	Not infected		Yes and confirmed	3	Yes	Agree	Neutral	Agree	Neutral	Neutral	Neutral	Disagree	Yes	Vaccines are used for government control	No	Yes	No	Yes	3	4	Unwilling to receive 4th dose (Please proceed to question 22a)	I'm worried about the side effects of the booster shots	4							
Timestamp	janushaa.nk@gmail.com	Agree	18-30 years old	Female	Indian	Single	Healthy adults	Diploma	No	Urban	No	No	Maybe	No	Yes	Yes and confirmed	5 days	Yes and confirmed	2	Yes	Agree	Agree	Agree	Neutral	Neutral	Neutral	Agree	Yes	Vaccines are dangerous/harmful and can cause serious side effects	Yes	Yes	No	Yes	3	3	Taken only 1st, 2nd, and 3rd dose but willing to receive 4th dose		4							
Timestamp	vijayapilachai@gmail.com	Agree	18-30 years old	Female	Indian	Single	Healthy adults	Undergraduate	No	Urban	No	No	Yes	Yes	No	Not infected		Not infected	1	Yes	Disagree	Disagree	Agree	Strongly agree	Strongly agree	Strongly agree	Strongly agree	No		Yes	Yes	Yes	Yes	5	5	Unwilling to receive 3rd dose (Please proceed to question 22a)	I'm worried about the side effects of the booster shots	4	4	Helps us monitor the current rate of infection among the patients					
Timestamp	lingeyshansenthilvasan@gmail.com	Agree	18-30 years old	Male	Indian	Single	Healthy adults	Diploma	No	Urban	No	No	Maybe	Yes	I don't know	I don't know		Yes and confirmed	3	Yes	Neutral	Agree	Agree	Agree	Agree	Agree	Agree	Yes	Vaccines are ineffective	Yes	Yes	No	Yes	3	3	Unwilling to receive 4th dose (Please proceed to question 22a)	I feel that the first and second doses are already sufficient to keep me protected from the virus	4							
Timestamp	grpriyasiny@gmail.com	Agree	18-30 years old	Female	Indian	Single	Healthy adults	Undergraduate	No	Urban	No	Yes	Yes	No	No	Not infected		I don't know	3	Yes	Agree	Agree	Agree	Disagree	Agree	Agree	Neutral	Yes	Vaccines are dangerous/harmful and can cause serious side effects	Yes	Yes	Yes	Yes	3	3	Received 1st, 2nd, and 3rd dose		4	3						
Timestamp	kavina9106@gmail.com	Agree	18-30 years old	Female	Indian	Single	Individuals older than 65 years	Undergraduate	No	Urban	No	No	Yes	No	No	Not infected		Not infected	4	Yes	Agree	Agree	Agree	Agree	Agree	Agree	Neutral	No		Yes	Yes	Yes	Yes	1	2	Received 1st, 2nd, and 3rd dose	I don't think it's necessary as the number of COVID-19 cases has gone down already	4	3	It gives me a sense of security knowing that the patient is not infected					
Timestamp	jxuanliew10@gmail.com	Agree	18-30 years old	Female	Chinese	Single	Healthy adults	Undergraduate	No	Urban	No	No	No		I don't know	I don't know		I don't know	1	No	Agree	Neutral	Neutral	Neutral	Agree	Agree	Neutral	No		Yes	Yes	Yes	Yes	3	1	Received 1st, 2nd, and 3rd dose		3							
Timestamp	logeswarankanawagi@gmail.com	Agree	31-40 years old	Male	Indian	Married	Children	Other	Yes	Urban	No	No	Yes	Yes	Yes	Not infected		Yes and confirmed	3	Yes	Neutral	Strongly disagree	Strongly disagree	Strongly disagree	Strongly disagree	Neutral	Neutral	Yes	Vaccines are dangerous/harmful and can cause serious side effects	Yes	Yes	Yes	Yes	3	3	Received 1st, 2nd, and 3rd dose	I don't think it's necessary as the number of COVID-19 cases has gone down already	2	3						
Timestamp	shahirah@pidc.edu.my	Agree	18-30 years old	Female	Malay	Single	Healthy adults	Secondary education	Yes	Urban	No	No	No		No	Not infected		I don't know	4	Yes	Strongly agree	Strongly agree	Strongly agree	Strongly agree	Strongly agree	Strongly agree	Neutral	Yes	Vaccines are dangerous/harmful and can cause serious side effects	Yes	Yes	Yes	Yes	5	3	Received 1st, 2nd, and 3rd dose	I'm worried about the side effects of the booster shots	5							
Timestamp	kadalarasi8@gmail.com	Agree	31-40 years old	Female	Indian	Married	Individuals older than 65 years	Postgraduate	No	Urban	No	Yes	Yes	No	Yes	Not infected		Not infected	1	Yes	Strongly disagree	Agree	Agree	Disagree	Agree	Neutral	Strongly disagree	No		Yes	Yes	Yes	Yes	5	5	Received 1st, 2nd, and 3rd dose	I'm worried about the side effects of the booster shots	4							
Timestamp	delhimani2012@gmail.com	Agree	41-50 years old	Male	Indian	Married	Children	Undergraduate	No	Urban	No	No	Yes	Yes	No	Not infected		I don't know	1	Yes	Agree	Agree	Agree	Agree	Agree	Agree	Neutral	No		Yes	Yes	Yes	Yes	5	5	Received 1st, 2nd, 3rd, and 4th dose	Good for immune	5							
Timestamp	senth49979@gmail.com	Agree	51-60 years old	Male	Indian	Married	Children	Postgraduate	No	Urban	No	No	Yes	Yes	No	Not infected	Not infected	Yes and confirmed	5	Yes	Disagree	Neutral	Neutral	Neutral	Disagree	Disagree	Disagree	No	Vaccines are used for government control	Yes	Yes	Yes	Yes	1	3	Received 1st and 2nd dose	I feel that the first and second dose is already sufficient to keep me protected from the virus	2	3	It gives me a sense of security knowing that the patient is not infected					
